# Antimicrobial blue light inactivates *Listeria monocytogenes* in diverse food systems with preserved physicochemical and sensory qualities

**DOI:** 10.1016/j.fochx.2026.104399

**Published:** 2026-09-03

**Authors:** Biao He, Yi Xu, Ruili Yang, Ying Zhang, Feng Zhu, Xueqin Cheng, Jianan Li, Jun-O Jin, Hui-Li Wang

**Affiliations:** a School of Food and Biological Engineering, Hefei University of Technology, Hefei 230000, China; b Department of Microbiology, University of Ulsan College of Medicine, Asan Medical Center 88, Songpa-gu, Seoul, Republic of Korea

**Keywords:** *Listeria monocytogenes*, Antimicrobial blue light, Orange juice, Surimi products, Sensory evaluation

## Abstract

*Listeria monocytogenes* is a major foodborne pathogen, capable of proliferating under refrigeration. Antimicrobial blue light (aBL) has emerged as a promising non-thermal decontamination strategy, yet its comprehensive mechanisms and compatibility with complex food systems remain incompletely characterized. Here, we demonstrate that aBL induces substantial killing of L. *monocytogenes* in a dose- and wavelength-dependent manner, and we identified the optimal combination of 420 nm and a fluence of 108 J/cm^2^, which achieved a maximum reduction of 3.8 log₁₀ CFU/mL (compared with 3–4 log reductions at other settings). Mechanistically, aBL suppresses free cells and biofilms by promoting ROS and autolysis, respectively. Transcriptomic profiling reveals significant upregulation of *spxA*, *kat*, and *recA* (log₂(fold change) > 1, padj <0.001), indicating strong activation of oxidative stress defense and DNA repair pathways. Subsequently, the food safety applicability of aBL was validated among liquid, solid, and packaged food formats, with 420 nm consistently eliciting superior efficacy while preserving sensory qualities of food products. Notably, in surimi, hardness, chewiness and cohesiveness were significantly enhanced, whereas springiness and adhesiveness remained unchanged. Importantly, aBL retained its bactericidal activity under refrigerated conditions, underscoring its practical value for cold-chain food storage. These findings establish aBL as a promising, scalable intervention strategy for L. *monocytogenes* decontamination across multiple food storage scenarios.

## Highlights

• aBL inactivates *Listeria monocytogenes* in a dose and wavelength dependent manner.
• aBL decontaminates listeria from multiple food matrices while maintaining quality.
• Divergent stress responsiveness arises from different wavelengths of blue light.
• aBL dissipates listeria biofilms through regulating autolysis in a stage-specific way.

## 1 Introduction

*Listeria monocytogenes* is a Gram-positive foodborne pathogen of major public health concern, causing listeriosis with high mortality in immunocompromised individuals, pregnant women, the elderly, and neonates (Radoshevich & Cossart, 2017; Koopmans et al., 2022; Gaspari et al. 2024). Unusually among foodborne pathogens, it grows at refrigeration temperatures (2–4 °C) and tolerates a wide range of pH and salt conditions, allowing it to persist throughout the cold chain and food supply (Wang et al., 2024; Ravindhiran et al., 2023; Vega-Sánchez et al. 2026). This persistence is compounded by phenotypic heterogeneity, including the emergence of small-colony variants with enhanced stress tolerance (Chan, Hussain, Hasan, et al., 2021). Among its contaminated foods, surimi-based products are typically stored under the same refrigerated conditions, which might favor the growth of L. *monocytogenes* (Ekonomou et al., 2020). Moreover, the existing preservation methods such as modified atmosphere packaging, chemical preservatives and heat, tend to compromise their defining textural and sensory qualities (Esteves et al., 2021). Curbing contamination without sacrificing quality therefore remains an unresolved challenge.

Existing control strategies, such as UV irradiation, pulsed light, cold plasma, and high-pressure processing, are constrained by limited penetration, equipment complexity, or adverse effects on food matrices (Wu et al. 2022; Lixandru et al. 2010; Olszewska et al., 2023), while aPDI (antimicrobial photodynamic inactivation) is promising but requires exogenous compounds as photosensitizers (Kashef & Hamblin, 2017). Comparatively, antimicrobial blue light (aBL, ∼400–470 nm) is a non-thermal, chemical-free alternative that generates cytotoxic ROS via photoexcitation of endogenous porphyrins (O'Donoghue et al., 2016; Yang et al., 2024), which is found effective against L. *monocytogenes* on select food surfaces (Olszewska et al., 2023; Rivera-Santiago & Diez-Gonzalez, 2025), and acts partly through the stress regulators SigB and Lmo0799 (Dorey et al., 2019; O'Donoghue et al., 2016). However, the comprehensive sub-cellular and molecular mechanisms of aBL against both planktonic cells and biofilms have not been systematically elucidated, and no study has examined aBL against L. *monocytogenes* in surimi, a matrix defined by gel strength and whiteness, under combined refrigerated, packaged/unpackaged conditions associated with real-world cold-chain logistics.

aBL has been found efficacious across liquid media and solid food surfaces (Ghate et al., 2013; Hur & Diez-Gonzalez, 2025; Xiao & Zhang, 2025). Its compatibility with food contact materials and transparency to packaging films theoretically supports its potential for in-package decontamination. However, despite these promising attributes, the application of aBL to surimi products has not been reported. Whether aBL can effectively suppress L. *monocytogenes* in surimi under practical storage conditions without damaging its quality, remains unknown.

We therefore hypothesize that aBL treatment at optimized wavelengths and doses will significantly reduce L. *monocytogenes* viability in both planktonic and biofilm states on surimi during refrigerated storage, without compromising product quality, and that the bactericidal effect is mediated by ROS-triggered oxidative damage and autolysis-related pathways. This hypothesis is raised based on the known photo-sensitivity of listerial porphyrins and the stress-regulatory networks identified in related species. To test this hypothesis, we systematically investigated the bactericidal efficacy and mechanisms of aBL against L. *monocytogenes* in multiple food application contexts, addressing the dose and wavelength dependent characteristics, antioxidative stress response pathways, autolysis-related biofilm suppression, as well as application to liquid/solid, unpackaged/packaged, room temperature/refrigeration scenarios. Given the growing interest of regulatory agencies such as the FDA and EFSA in novel light-based technologies for food safety, aBL's residue-free and packaging-compatible nature aligns well with the current framework for “novel food processing” approvals, thereby enabling the discovery here a promising step toward practical application.

## 2 Materials and methods

### 2.1 Bacterial strain and culture conditions

*Listeria monocytogenes* (BNCC185986) was obtained from BeNa Culture Collection (Zhengzhou, China) and stored at −80 °C. The strain originates from a clinical isolate (serotype 1/2a, as certified by the supplier). For experiments, bacteria were inoculated at 1% (*v*/v) into Brain Heart Infusion (BHI) broth (Huankai, Guangzhou, China) and cultured at 37 °C with shaking at 180 rpm until the mid-logarithmic phase was reached (OD_600_ value of 10^7^–10^8^ CFU/mL). Cells were harvested by centrifugation (8000 *g*, 3 min), washed twice with sterile phosphate-buffered saline, and resuspended in PBS to a final density of ∼10^7^ CFU/mL for subsequent experiments.

### 2.2 aBL irradiation

Antimicrobial blue light (aBL) was delivered using a custom-built LED irradiation system housed in a closed chamber to exclude ambient light interference. LEDs of five wavelengths (420, 430, 440, 450, and 460 nm) were used in this study. The LED irradiation system (model DB, Ningbo Jiadeng Electronics Co., Ltd., China) has a spectral bandwidth (FWHM) of 15–20 nm. For all illumination experiments, the LED was positioned 15 cm above the sample surface in a 6-well plate. This distance was chosen to ensure a uniform illuminated area covering the entire well plate; uniformity was verified by measuring irradiance at nine grid points using a power meter. The irradiance was calibrated to 20 mW/cm^2^ using a laser power meter (VLP-2000, Beijing, China), yielding light doses of 0, 18, 36, 72, 108, and 144 J/cm^2^ for durations of 0, 15, 30, 60, 90, and 120 min, respectively. During irradiation at room temperature, the sample temperature was continuously monitored using an infrared thermometer (Fluke 62 MAX, USA) and remained within 25 ± 1 °C; no active cooling was required. To ensure homogeneous exposure, the 6-well plate was gently agitated manually every 15 min throughout the treatment. For experiments conducted at 4 °C, the entire irradiation device was placed in a refrigerator to prevent sample heating and to simulate refrigerated storage conditions. The bacterial enumeration was carried out by serial plate counting. Bacterial viability was expressed as normalized survival rate relative to untreated controls.

### 2.3 LIVE/DEAD fluorescence staining

Membrane integrity was assessed using propidium iodide (PI) staining, and bacterial viability was evaluated using the BacLight™ LIVE/DEAD Bacterial Viability Kit (Molecular Probes, Invitrogen, USA), which employs SYTO 9 (green, live cells) and PI (red, membrane-compromised cells). Following aBL treatment, bacterial cells were washed and resuspended in sterile PBS. For PI staining, 3 μL of PI stock solution (1 mg/mL in DMSO, approximately 1.5 mM) was added to 1 mL of bacterial suspension, yielding a final PI concentration of approximately 4.5 μM, and incubated at room temperature for 25 min in the dark. For LIVE/DEAD dual staining, SYTO 9 stock (5 mM in DMSO) and PI stock (1 mg/mL, ∼1.5 mM) were thawed on ice, mixed in equal volumes (1:1), and 3 μL of this mixture was added to 1 mL of bacterial suspension—resulting in final concentrations of approximately 7.5 μM SYTO 9 and 2.25 μM PI (since mixing equal volumes halves each stock concentration before the 3-μL addition)—followed by incubation at room temperature for 25 min in the dark. After staining, washing, and quenching, 10 μL of the stained cell suspension was mounted on a glass slide. Samples were imaged using a confocal laser scanning microscope (ZEISS LSM880 with Airyscan, Germany) with excitation channels at 488 nm (SYTO 9) and 543 nm (PI). Fluorescence intensity was quantified using ImageJ software (NIH, USA).

### 2.4 Intracellular ROS measurement and antioxidant enzyme (SOD/CAT) activity assay

Intracellular reactive oxygen species (ROS) were detected using the fluorescent probe DCFH-DA (2′,7′-dichlorofluorescein diacetate; Maokang Biotechnology, Shanghai, China). After aBL treatment, 3 μL of DCFH-DA was added to 1 mL of bacterial suspension and incubated at 37 °C for 30 min in the dark with gentle mixing every 5 min. Following the subsequent steps of washing, quenching, and mounting, fluorescence was imaged using confocal microscopy (ZEISS LSM880 with Airyscan, Germany) at the 488 nm excitation channel, and ROS levels were quantified using ImageJ software.

To assess the antioxidant enzymatic response to aBL, catalase (CAT) and superoxide dismutase (SOD) activities were measured using commercial activity assay kits (Solarbio Science & Technology, Beijing, China). Following 20 min of aBL irradiation, bacterial cells were harvested by centrifugation and the supernatant discarded. Cells were resuspended in 1 mL of the kit-supplied extraction buffer and lysed by sonication (200 W, 3 s pulses with 10 s intervals, repeated 30 times), followed by centrifugation at 8000 *g* for 10 min at 4 °C; the resulting supernatant was kept on ice for immediate analysis. CAT activity was calculated as CAT (U/10^4^ cells) = 1.529 × ΔA × F, where ΔA is the difference in absorbance at 240 nm measured 5 s (A1) and 1 min 5 s (A2) after substrate addition, and F is the sample dilution factor. SOD activity was calculated from the percentage inhibition of the chromogenic reaction at 450 nm relative to a blank, according to SOD activity (U/10^4^ cells) = [10 × inhibition% + (1 − inhibition%)] / N × F, where N is the total bacterial cell number (×10^4^) and F is the dilution factor.

### 2.5 Intracellular DNA and protein leakage assay

Following aBL treatment, bacterial suspensions were centrifuged (8000 *g*, 3 min) and the supernatants were filtered through 0.22 μm membranes. Aliquots of 200 μL were transferred to 96-well plates, and absorbance was measured at 260 nm (DNA) and 280 nm (protein) using a microplate reader (Infinite 200 Pro, Tecan, Switzerland).

### 2.6 Scanning electron microscopy (SEM)

After aBL treatment, the harvested cells were fixed in 2.5% glutaraldehyde at room temperature for 1.5 h, followed by overnight fixation at 4 °C. Cells were then washed three times with PBS and dehydrated through a graded ethanol series (10%, 30%, 50%, 70%, 90%, and 100%), with 15 min incubation at each interval. After air-drying, samples were sputter-coated with gold and examined using a thermal field emission scanning electron microscope (Gemini 500, Carl Zeiss, Germany).

### 2.7 Biofilm quantification

Biofilm quantification was performed as described previously with minor modifications (Stiefel et al., 2016). Briefly, *L. monocytogenes* suspensions (∼10^7^ CFU/mL) were treated with aBL and subsequently resuspended in fresh BHI broth. Aliquots of 200 μL were added to 96-well plates and incubated at 37 °C for 12, 24, 36, or 48 h. After incubation, wells were washed three times with sterile PBS and air-dried at 60 °C for 90 min. Biofilms were stained with 0.1% crystal violet (200 μL/well) for 20 min in the dark, rinsed with PBS, and air-dried. Plates were imaged using a fluorescence microscope (Eclipse Ti2, Nikon, Japan) in bright-field mode. Biofilm biomass was quantified by dissolving the stain in 200 μL of 40% acetic acid and measuring OD_590_ using a microplate reader.

### 2.8 Autolysis assay

Following aBL treatment, bacterial cells were washed twice and resuspended in 50 mM Tris-HCl buffer (pH 7.0) to an OD_600_ of 0.5–0.8. The suspensions were incubated at 37 °C and OD_600_ was measured at 12, 24, 36, and 48 h. The autolysis ratio was calculated as the proportional decrease in OD_600_ relative to the initial value at each time point.

### 2.9 RT-qPCR analysis

Total RNA was extracted from L. *monocytogenes* following aBL treatment using RNAprep Pure Cell / Bacteria Kit (Tiangen, Beijing, China). RNA quality and concentration were assessed by NanoDrop spectrophotometry. cDNA was synthesized by reverse transcription using TransScript® All-in-One First-Strand cDNA Synthesis SuperMix (TransGen Biotech, Beijing, China). Quantitative PCR was performed on a LightCycler 96 system (Roche, Basel, Switzerland) using PerfectStart Green qPCR SuperMix (TransGen Biotech, Beijing China). Gene expression was normalized to the 16S rRNA reference gene, and relative expression levels were calculated using the 2^−ΔΔCt^ method.

### 2.10 Transcriptome sequencing and analysis

Total RNA was extracted from L. *monocytogenes* treated with 420 nm aBL at 108 J/cm^2^ (*n* = 3). Strand-specific RNA libraries were constructed by NEBNext Ultra II RNA Library Prep Kit (NEB, USA) and sequenced on the Illumina NovaSeq platform (Illumina, San Diego, USA) with paired-end 150 bp reads. RNA integrity was verified using an Agilent 2100 Bioanalyzer (Agilent Technologies, CA, USA), and all samples yielded RIN values ≥7.0, confirming high-quality RNA suitable for sequencing.

Raw reads were quality-filtered and mapped to the L. *monocytogenes* reference genome using Bowtie2 (version 2.5.4). Differential expression analysis was performed using DESeq2, with differentially expressed genes (DEGs) defined by |log_2_(fold change)| > 1 and adjusted *p*-value (padj) < 0.05 (FDR is used here to ensure rigorous control of Type I errors). Gene Ontology (GO) and KEGG pathway enrichment analyses were conducted using clusterProfiler (4.8.1). Principal component analysis (PCA) was performed in R. Gene interaction network analysis was performed using the STRING database (https://string-db.org/), and graphically presented by Cytoscape (https://cytoscape.org/). All libraries were sequenced in a single batch on the same flow cell, thus batch-effect correction was not applicable in this study. The sequencing data is deposited in Genbank and can be accessed with accession no. PRJNA1501710.

### 2.11 Orange juice preparation and aBL treatment

Fresh oranges of the ‘Navel’ variety (*Citrus sinensis*) at commercial maturity (uniform orange color and firmness) and juicing equipment were pre-cooled at 4 °C for 3 h. Juice was extracted, filtered through gauze, and centrifuged (800 *g*, 10 min, 4 °C) to obtain the clarified supernatant. The juice yield was approximately 420 mL per kg of oranges. The centrifugation speed and time were optimized by preliminary trials to remove pulp and cloud without affecting soluble solids content. *L. monocytogenes* was inoculated into 2 mL aliquots of fresh orange juice to a final concentration of ∼10^6^ CFU/mL. Samples were transferred to 6-well plates and exposed to 420 nm or 440 nm aBL at 108 J/cm^2^ at either room temperature (RT) or 4 °C. Following treatment, all samples were stored at the respective temperatures for 48 h prior to analysis.

### 2.12 Physicochemical and sensory quality of orange juice

After 48 h storage, the following quality parameters were assessed:

pH was measured using a digital pH meter (PHS-3E, Yile Science Instrument, Shanghai, China). Soluble solids content was determined using a handheld refractometer (Delixi Electric, Hangzhou, China) and expressed in °Brix. Total polyphenol content was determined by the Folin method (Al-Ogaili et al., 2023). Results were expressed as gallic acid equivalents (mg GAE/L) using a gallic acid standard curve.

Polyphenol oxidase (PPO) activity was measured in a 96-well plate by combining 100 μL phosphate-citrate buffer (0.05 mol/L, pH 6.8), 50 μL sample, and 100 μL freshly prepared 0.1 mol/L catechol solution (Yuanye, Shanghai, China). Absorbance at 420 nm was recorded every 30 s for 3 min. PPO activity was defined as a change of 0.001 absorbance units per mL per min (Manzocco et al., 2017).

Sensory evaluation was performed by a panel of 16 trained assessors (10 females, 6 males, aged 22–30) using a 10-point hedonic scale (1 = poor, 10 = excellent; minimum acceptable score = 5). Prior to testing, all assessors underwent two 1-h training sessions covering the recognition of orange juice sensory attributes (color, odor, taste, cloudiness) and the use of the scale; reference standards were provided. To evaluate panel agreement, Kendall's coefficient of concordance (W) was calculated for each attribute. Color, odor, taste, cloudiness, and global sensory quality were scored with untreated fresh orange juice as the reference. Results were presented as radar charts. In addition, a consumer acceptance test was conducted with 25 untrained consumers using the same hedonic scale to assess overall liking. To further test whether any treatment-induced sensory difference was perceptible, a triangle discrimination test was additionally performed with the same 16 trained assessors, comparing the untreated control against each of the Mock and aBL-treated samples under both storage conditions.

**Sensory Evaluation Ethics information:** ethical permission was not required here to carry out a sensory evaluation. We confirm that the appropriate protocols for protecting the rights and privacy of all participants were utilized during the execution of the research.

**Sensory Evaluation Consent information:** we confirm that the participants gave their consent to take part in the sensory study and use their information.

### 2.13 Surimi sample preparation and aBL treatment

Commercially purchased surimi products were stored at −20 °C and thawed at room temperature before use. This product is Jingzhou Fish Cake (Hubei, China). According to the manufacturer's label, the ingredients are fresh freshwater fish, eggs, ginger, scallions, pork, starch, edible salt, and drinking water. To ensure homogeneity across samples, all slices (approximately 3 × 3 × 0.7 cm^3^) were taken from the same production batch and randomly assigned to treatment groups. Fish cake slices (approximately 3 × 3 × 0.7 cm^3^) were surface-sterilized by immersion in 75% (*v*/v) ethanol and air-dried in a laminar flow hood. *L. monocytogenes* suspension was inoculated onto the surface of each slice to achieve a final concentration of ∼10^6^ CFU/g, followed by 30 min adhesion in the laminar flow hood. For packaged surimi experiments, sterilized slices were placed in food-grade packaging bags (5 × 7 cm^2^), and bacterial suspension was inoculated onto the outer surface of the bags to the same concentration. The bags were made of polyethylene (PE) with a thickness of 0.08 mm and a light transmittance at 420 nm of 86% (measured by UV–Vis spectrophotometry). Inoculated samples were exposed to 420 nm or 440 nm aBL at 108 J/cm^2^ at RT or 4 °C. After irradiation, all samples (aBL-treated inoculated, untreated inoculated [Mock], and untreated uninoculated [Ctrl]) were stored at the respective temperatures for 48 h.

### 2.14 Bacterial enumeration on surimi and packaging surfaces

For surimi slices, bacterial counts were determined after homogenization of the slice in sterile PBS using a homogenizer (FJ200-SH, ZhiNiuCheBo Instrument, Shanghai, China) for 3 min, followed by serial dilution and plate counting. For packaging bag surfaces, sterile cotton swabs were used to uniformly wipe the bag surface, and swab heads were transferred into sterile PBS for vortexing. The resulting suspension was serially diluted, with colonies subsequently enumerated.

### 2.15 Physicochemical and textural quality of surimi

Surimi slices were homogenized in sterile PBS after 48 h storage. The following parameters were assessed on the homogenate or intact slices:

pH was measured according to similar procedure with orange juice.

Lipid oxidation was determined by the thiobarbituric acid reactive substances (TBARS) assay as described previously (Goethals et al., 2020).

Protein carbonyl content was determined by the DNPH (2,4-dinitrophenylhydrazine) method as described previously (Sun et al., 2025).

Texture profile analysis (TPA) was performed using a TA.XTplusC Texture Analyser (Stable Micro Systems, Surrey, UK) equipped with a P50 cylindrical probe. Parameters: pre-test and post-test speed 2.0 mm/s; test speed 1.0 mm/s; compression ratio 30%; trigger force 5.0 g; inter-compression interval 6 s. Chewiness, cohesiveness, and hardness were recorded.

Sensory evaluation was conducted using the same trained panel (16 assessors) and the same 10-point hedonic scale as for orange juice, with attributes including color, odor and texture. Panel agreement was again assessed by Kendall's W. In addition, a consumer acceptance test was conducted with 25 untrained consumers. A triangle discrimination test was likewise performed for both unpackaged and packaged surimi.

### 2.16 Statistical analysis

All experiments were performed in triplicate and data are expressed as mean ± standard error of the mean (SEM). Sample size was determined based on a target statistical power (> 0.8, α = 0.05) from preliminary variance estimates. Statistical analyses were performed using GraphPad Prism (version 10.1.2, GraphPad Software, San Diego, USA) and SPSS (version 27.0, IBM, New York, USA). One-way ANOVA with Tukey's honestly significant difference (HSD) *post-hoc* test was used for multiple pairwise comparisons. Radar charts were generated using Origin (version 2024, Northampton, USA). A *p*-value <0.05 was considered statistically significant. Dose-response curves for EC₅₀/EC₉₀ determination were fitted using a four-parameter logistic (Hill) model in GraphPad Prism.

## 3 Results

### 3.1 aBL exerts dose- and wavelength-dependent bactericidal effects on L. monocytogenes

**Fig. 1:**
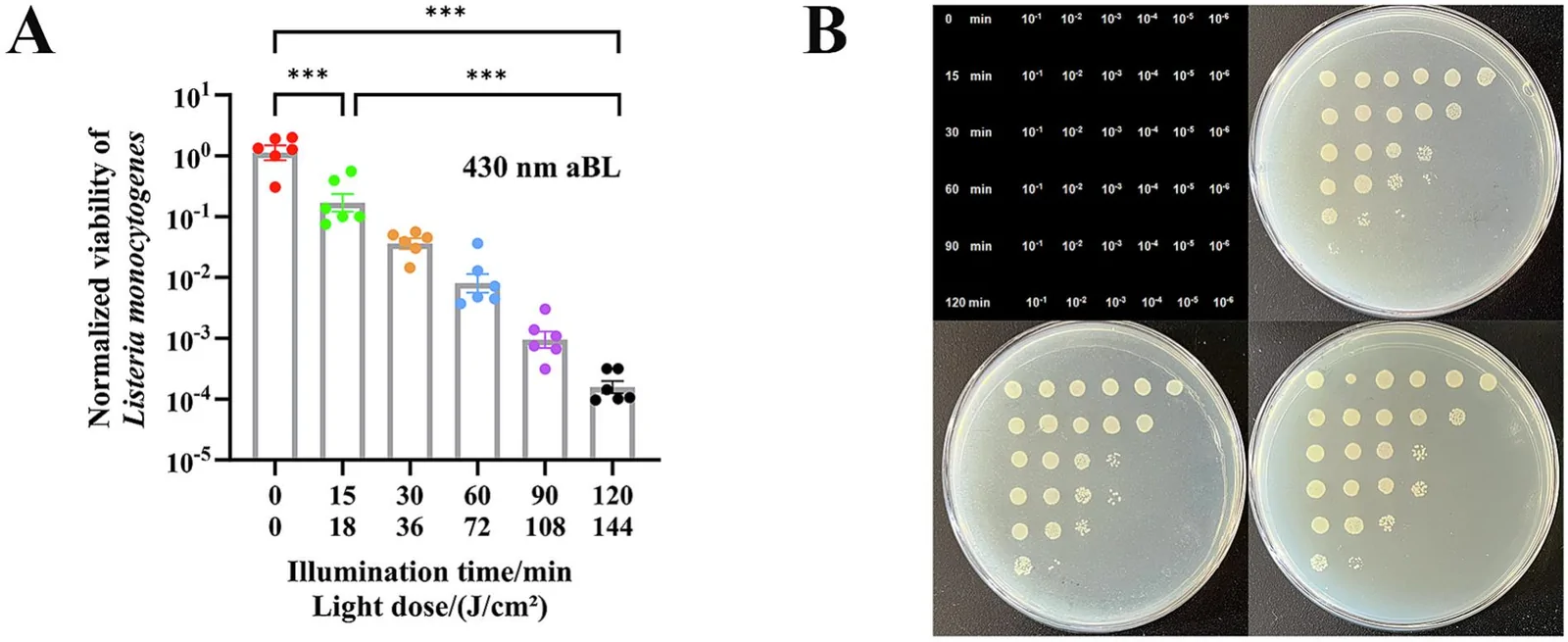
aBL exerts bactericidal effects on *Listeria monocytogenes* under varying illumination. (A) Normalized viability of L. *monocytogenes* following exposure to 430 nm aBL at an irradiance of 20 mW/cm^2^ for 0, 15, 30, 60, 90, and 120 min, corresponding to light doses of 0, 18, 36, 72, 108, and 144 J/cm^2^, respectively. Data are presented as mean ± SEM from three independent experiments. Statistical significance was determined by one-way ANOVA (***p < 0.001). (B) Representative photographs of spot assay plates showing the colony-forming units of L. *monocytogenes* at serial dilutions (10^−1^ to 10^−6^) following aBL treatment at the indicated time points. The aBL irradiation setup and LED source plate are shown in Fig. S1.

The bactericidal efficacy of antimicrobial blue light (aBL) against *Listeria monocytogenes* needs to be determined first. Accordingly, an irradiation device was customized, in which an LED source plate was positioned 15 cm above a 6-well plate containing bacterial samples within a closed box (Fig. S1 A, B). Since 430 nm is an approximately median wavelength within the blue light range of 400–470 nm, it was first adopted to assess the bactericidal effect of aBL on *Listeria monocytogenes*. As shown in Fig. 1A and B, aBL treatment significantly decreased the viability of L. *monocytogenes* in a dose-dependent manner, reaching a maximum reduction of approximately 4 logs when the irradiation of 144 J/cm^2^ was applied.

A four-parameter logistic nonlinear regression model was applied to quantitatively assess the wavelength-dependent bactericidal efficacy of antimicrobial blue light (aBL) against *Listeria monocytogenes*. The results revealed obvious differences in the antimicrobial activity of aBL across different wavelengths (Table S1). 420 nm aBL showed the strongest bactericidal effect, with EC₅₀ and EC₉₀ values of 4.2 J/cm^2^ and 7.5 J/cm^2^, respectively. The precisely calibrated EC₅₀ and EC₉₀ of 430 nm aBL were 10.6 J/cm^2^ (95% CI: 8.9–12.7 J/cm^2^) and 18.3 J/cm^2^ (95% CI: 16.2–20.8 J/cm^2^). The bactericidal efficacy gradually decreased with increasing wavelength. 460 nm and 450 nm aBL exhibited moderate antibacterial activity, with EC₉₀ values of 39.0 J/cm^2^ and 50.0 J/cm^2^. Among all tested groups, 440 nm aBL had the weakest inactivation performance, requiring a fluence of 108.0 J/cm^2^ to achieve 90% bacterial inactivation.

**Fig. 2:**
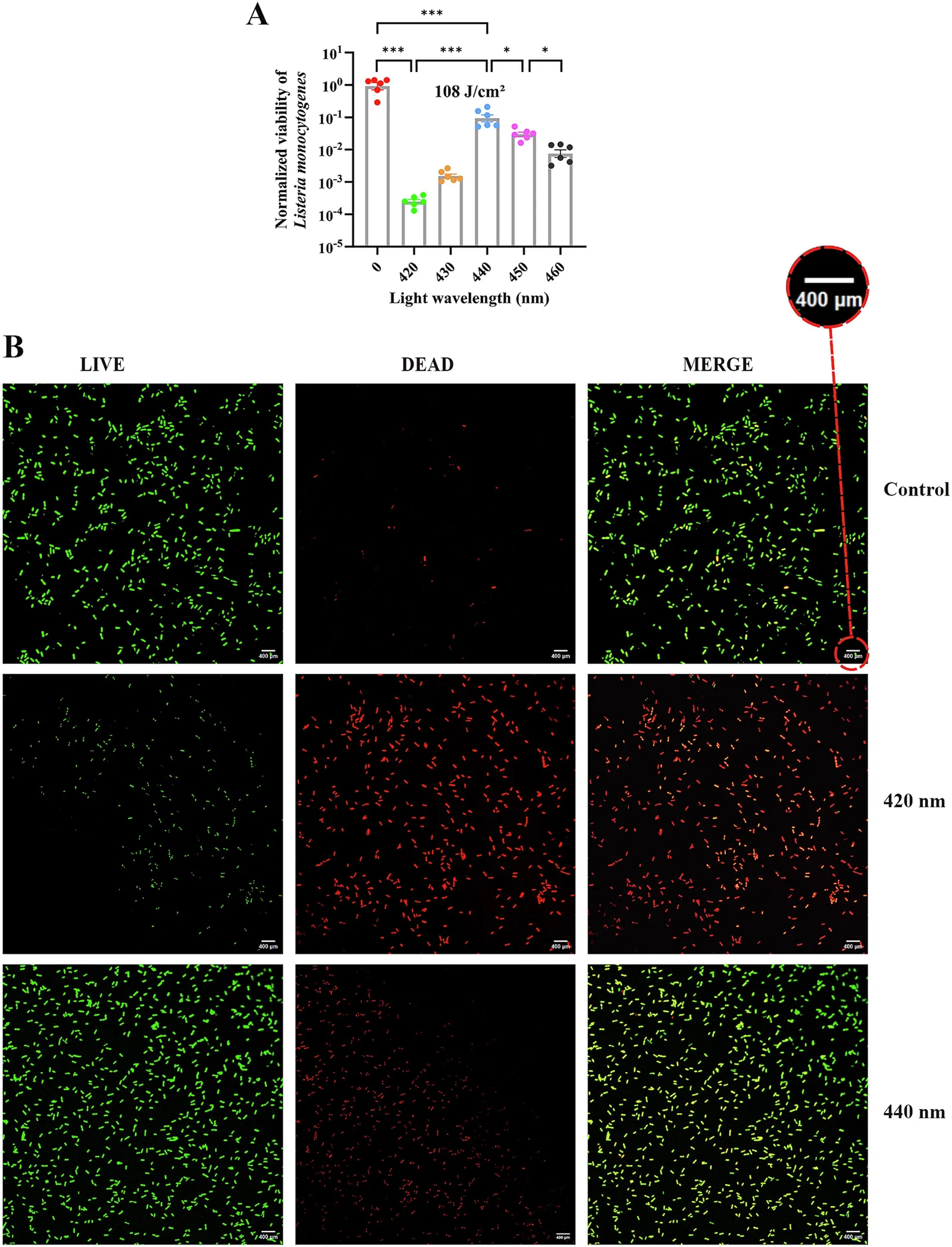
The antimicrobial effect of aBL on *Listeria monocytogenes* is wavelength-dependent. (A) Normalized viability of L. *monocytogenes* following exposure to aBL at different wavelengths (420, 430, 440, 450, and 460 nm) at a fixed light dose of 108 J/cm^2^. Data are presented as mean ± SEM from three independent experiments. Statistical significance was determined by one-way ANOVA (*p < 0.05, ***p < 0.001). Representative spot-assay photographs underlying this panel are shown in Fig. S2. (B) Representative confocal laser scanning microscopy images of L. *monocytogenes* stained with the BacLight LIVE/DEAD kit following treatment with 420 nm or 440 nm aBL at 108 J/cm^2^, with untreated cells serving as the control. Live cells are shown in green and dead cells in red. Scale bar = 400 μm. (For interpretation of the references to color in this figure legend, the reader is referred to the web version of this article.)

To further determine the optimal bactericidal wavelength, *L. monocytogenes* was exposed to aBL at different wavelengths (420, 430, 440, 450, and 460 nm) at a fixed light dose of 108 J/cm^2^. The results demonstrated that the antimicrobial efficacy varied considerably depending on the wavelength used (Fig. 2A; Fig. S2). Among all wavelengths tested, 420 nm aBL exhibited the strongest bactericidal effect, reducing the bacterial survival to approximately 10^−4^, which was significantly greater than all other wavelengths (*p* < 0.001). Dose-response fitting using a four-parameter logistic model (Table S1) confirmed this ranking quantitatively, yielding EC₅₀/EC₉₀ values of 4.2/7.5 J/cm^2^ for 420 nm and 10.6/18.3 J/cm^2^ for 430 nm, versus substantially higher values of 52.0/108.0 J/cm^2^ for 440 nm, 28.0/50.0 J/cm^2^ for 450 nm, and 22.0/39.0 J/cm^2^ for 460 nm. Notably, 430 nm also showed potent killing activity, whereas 440, 450, and 460 nm 460 nm displayed only modest inhibition. Quantitatively, at 108 J/cm^2^, 420 nm reduced viable counts by 3.8 log₁₀ CFU/mL, which was approximately 2.3-fold more effective than 440 nm (1.7 log₁₀ reduction) under the same conditions. These findings were further confirmed by LIVE/DEAD fluorescence staining (Fig. 2B). Collectively, aBL exerts dose- and wavelength-dependent bactericidal effects against L. *monocytogenes*. These findings, however, were obtained using a single reference strain (BNCC185986), and further validation with a diverse panel of strains is warranted to establish broader generalizability.

### 3.2 aBL disrupts listeria membrane integrity and induces intracellular content leakage

**Fig. 3:**
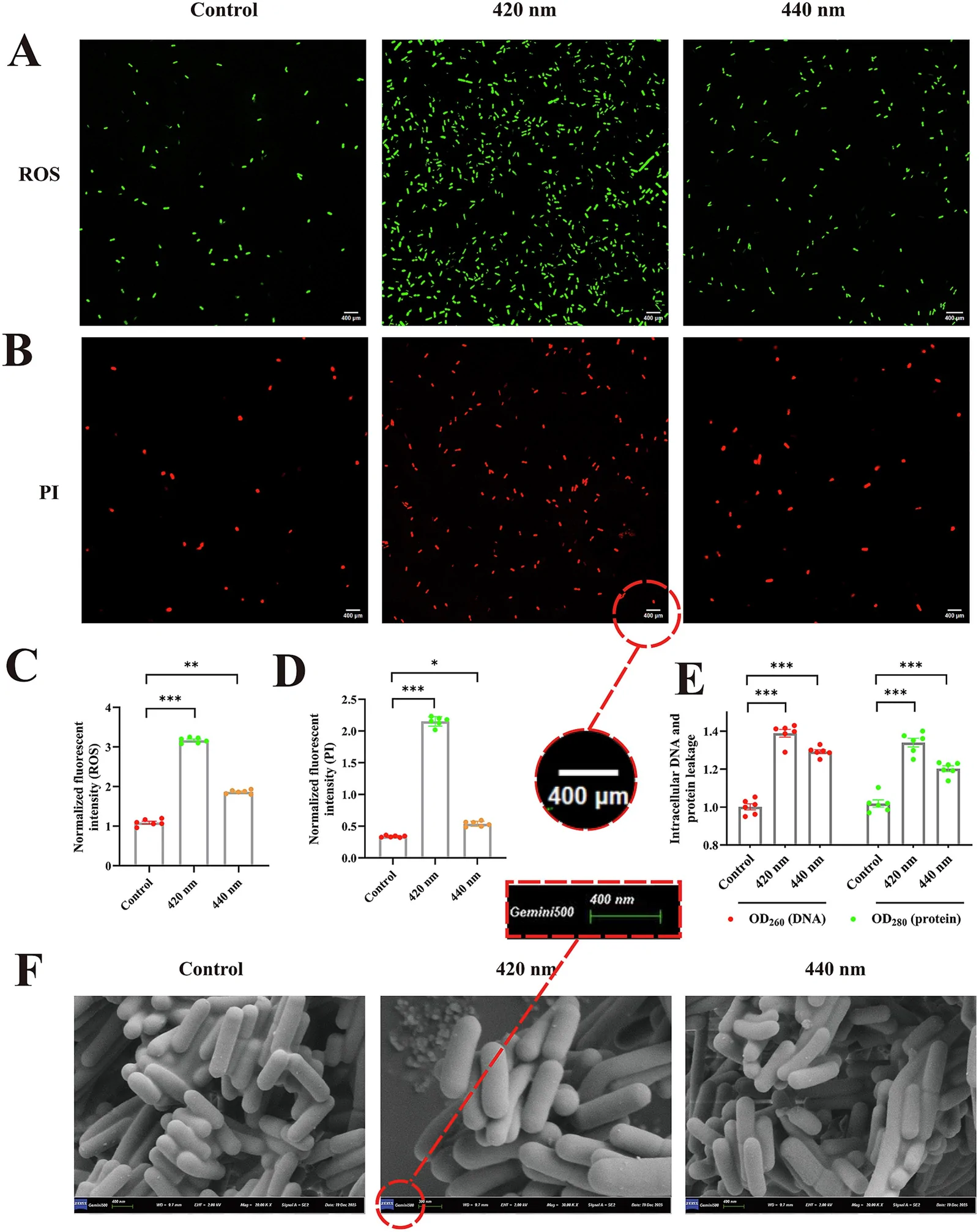
aBL damages bacterial surface structure and releases the intracellular DNAs. (A, B) Representative confocal laser scanning microscopy images of L. *monocytogenes* stained with DCFH-DA for intracellular ROS detection (A, green) and propidium iodide (PI) for membrane integrity assessment (B, red) following treatment with 420 nm or 440 nm aBL at 108 J/cm^2^. Scale bar = 400 μm. (C, D) Quantification of normalized fluorescent intensity of ROS (C) and PI (D) signals across the three groups. (E) Leakage of intracellular DNA and protein from L. *monocytogenes* following aBL treatment at 108 J/cm^2^, as measured by absorbance at OD₂₆₀ (DNA, red) and OD₂₈₀ (protein, green). (F) Representative scanning electron microscopy images of L. *monocytogenes* following treatment with 420 nm or 440 nm aBL at 108 J/cm^2^, showing aBL-induced morphological changes in bacterial surface structure. Data are presented as mean ± SEM from three independent experiments. Statistical significance was determined by one-way ANOVA (*p < 0.05, **p < 0.01, ***p < 0.001). (For interpretation of the references to color in this figure legend, the reader is referred to the web version of this article.)

In terms of the sub-cellular mechanisms underlying aBL-mediated inhibition of L. *monocytogenes*, we investigated the generation of reactive oxygen species (ROS) and the resultant damage to the bacterial membrane integrity. Using DCFH-DA fluorescence staining, aBL treatment markedly elevated intracellular ROS levels in L. *monocytogenes* (Fig. 3A). Consistently, propidium iodide (PI) staining, which selectively penetrates cells with compromised membranes, showed a robust increase in PI-positive bacteria following aBL treatment (Fig. 3B), indicative of severe membrane disruption. These results were supported by quantification analysis (Fig. 3C, D). Furthermore, to determine whether bacteria mount an antioxidant defense against this aBL-induced oxidative stress, we measured the activities of superoxide dismutase (SOD) and catalase (CAT). Both enzyme activities were significantly elevated following aBL treatment, with a greater increase observed under 420 nm than 440 nm irradiation (Fig. S3A, B), indicating that L. *monocytogenes* activates its antioxidant defense system in response to aBL-generated ROS.

As a consequence of membrane disruption, significant leakage of intracellular contents was detected in aBL-treated bacteria. Measurements of the supernatant at OD_260_ and OD_280_ revealed that both intracellular DNA and protein concentrations were significantly boosted in the 420 nm and 440 nm treatment groups relative to the control (*p* < 0.001), with 420 nm eliciting a greater degree of leakage (Fig. 3E). Scanning electron microscopy (SEM) further corroborated these findings at the morphological level, with aBL-treated cells exhibiting severe structural deformation, including surface wrinkling, membrane blebbing, and cell collapse, during which more pronounced damage was observed in the 420 nm group (Fig. 3F). Taken together, aBL triggers ROS-mediated oxidative damage, which might be associated with disruption of the bacterial cell membrane and leakage of intracellular macromolecules, which may underlie the consequent cell death.

### 3.3 aBL inhibits biofilm formation and promotes autolysis of L. *monocytogenes*

**Fig. 4:**
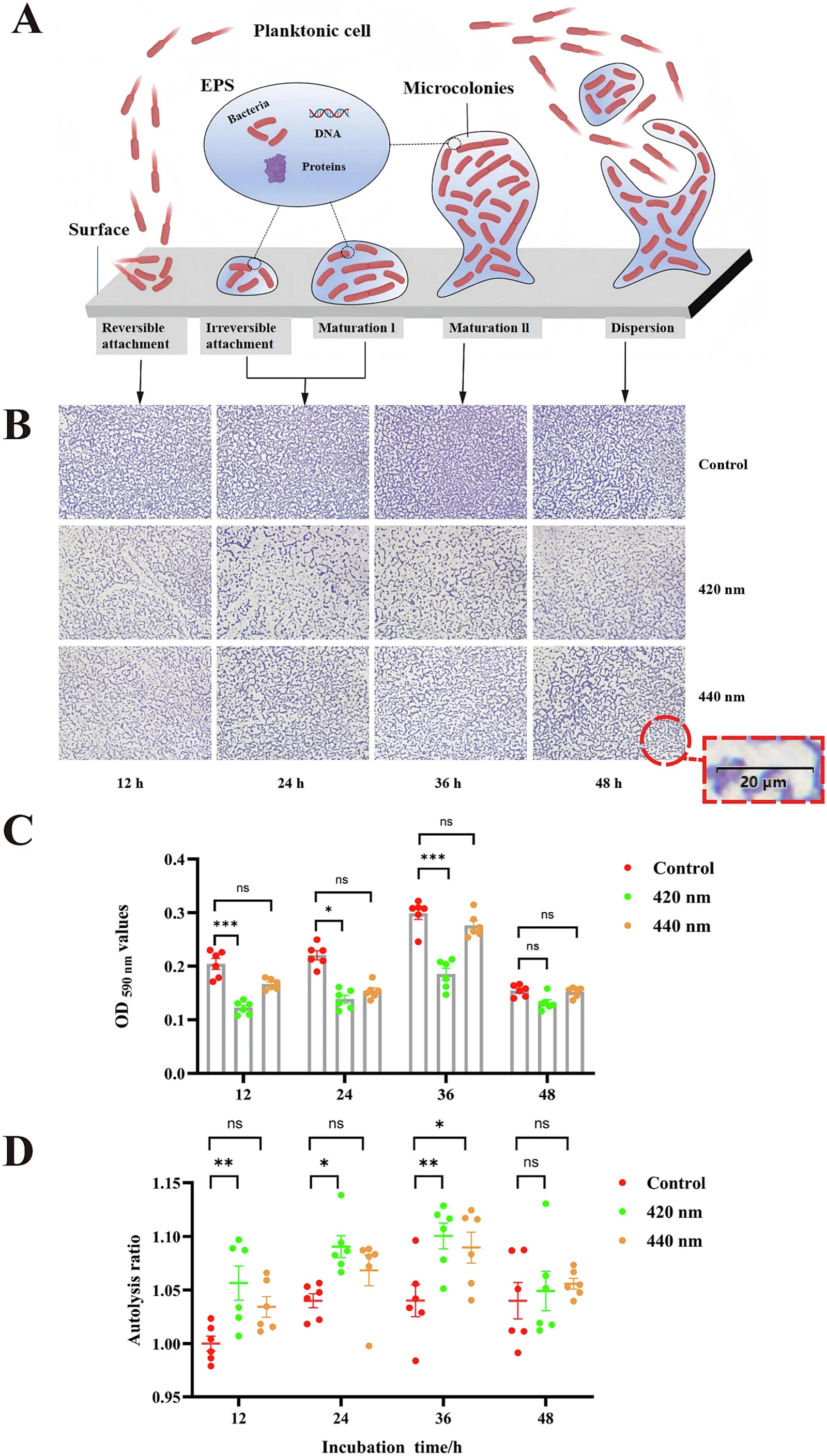
aBL disrupts biofilm formation of *Listeria monocytogenes*. (A) Schematic illustration of the bacterial biofilm formation process, depicting the sequential stages from planktonic cells to reversible attachment, irreversible attachment, maturation I, maturation II, and dispersion, with the biofilm matrix components including extracellular polymeric substances (EPS), DNA, and proteins indicated. (B) Representative fluorescence microscopy images of crystal violet-stained L. *monocytogenes* biofilms following treatment with 420 nm or 440 nm aBL at 108 J/cm^2^ at 12, 24, 36, and 48 h of incubation, with untreated cells serving as the control. Scale bar = 20 μm. (C) Quantification of biofilm biomass by measuring absorbance at OD₅₉₀ nm at the indicated time points across the three groups. (D) Autolysis ratio of L. *monocytogenes* following aBL treatment at the indicated time points, expressed as the ratio of the decrease in optical density during the incubation period. Data are presented as mean ± SEM from three independent experiments. Statistical significance was determined by one-way ANOVA (ns, not significant; *p < 0.05; **p < 0.01; ***p < 0.001). (For interpretation of the references to color in this figure legend, the reader is referred to the web version of this article.)

Given that L. *monocytogenes* readily forms biofilms on food contact surfaces (Puranen et al., 2021), a key assembly contributing to resilient contamination, we next investigated whether aBL could exert detrimental effects on listeria biofilms. As illustrated in Fig. 4A, bacterial biofilm development proceeds through sequential stages, from reversible and irreversible attachment to maturation and dispersion, accompanied by the secretion of extracellular polymeric substances (EPS) containing DNA and proteins. Crystal violet staining revealed that aBL treatment visibly weakened biofilm density at multiple time points compared to untreated controls, corresponding to the stages of attachment and maturation (Fig. 4B). Quantification of biofilm biomass showed that 420 nm aBL treatment led to a markedly compromised biofilm at 12 h (*p* < 0.001) and 36 h (*p* < 0.001), while 440 nm posed no statistically significant effect at most time points (Fig. 4C). These results suggest that 420 nm aBL is selectively effective at interfering with early attachment and particularly biofilm maturation stages.

**Fig. 5:**
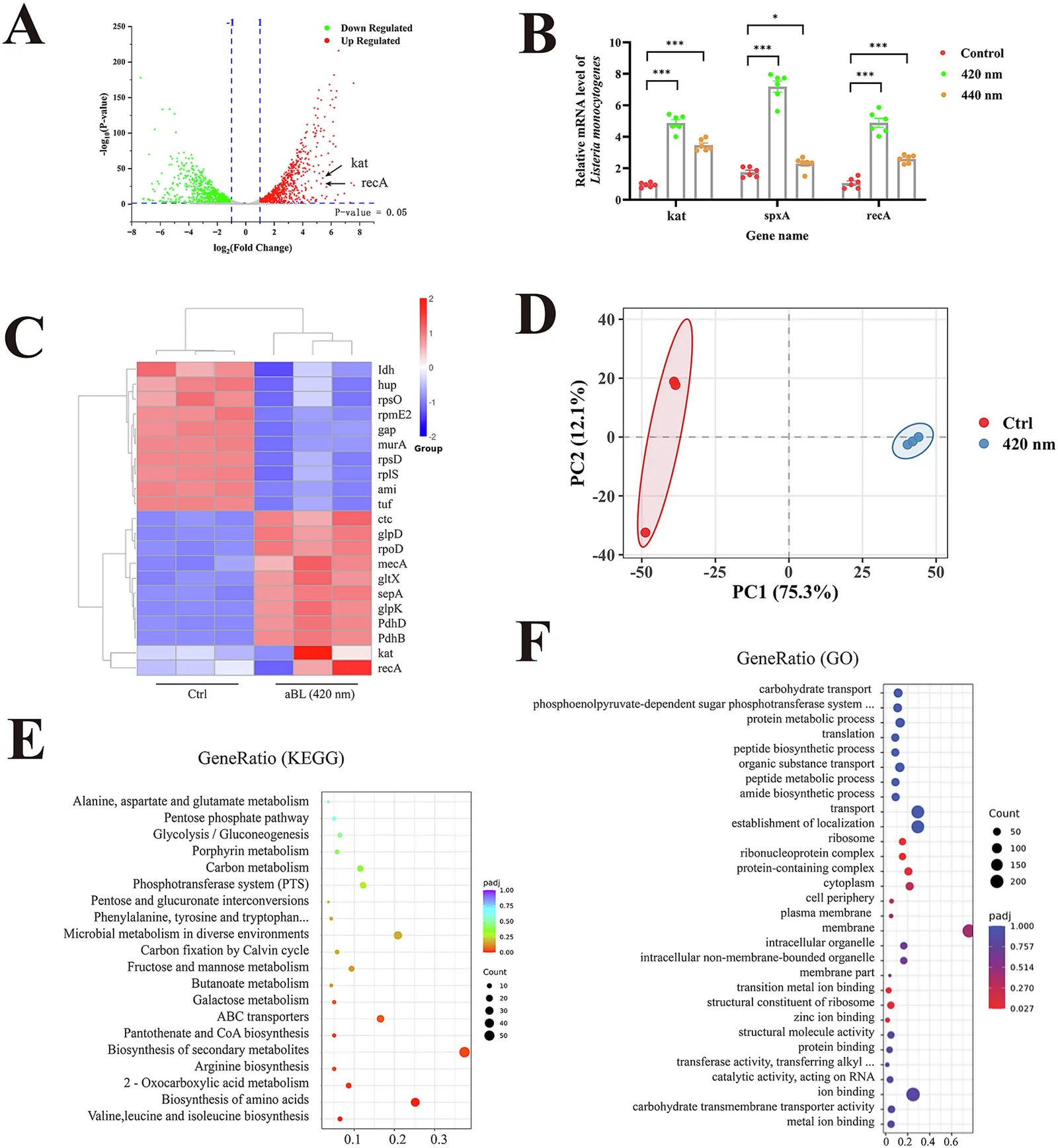
Transcriptomics profiles the molecular response of *Listeria monocytogenes* treated with aBL. (A) Volcano plot displaying the differentially expressed genes (DEGs) between control (Ctrl) and aBL-treated L. *monocytogenes*, with upregulated genes shown in red and downregulated genes in green. Key genes including *spxA*, *Kat*, and *recA* are labeled. DEGs were identified using the criteria of |log2(fold change)| > 1 and padj <0.05. (B) Relative mRNA levels of *Kat*, *spxA*, and *recA* in L. *monocytogenes* following treatment with 420 nm or 440 nm aBL at 108 J/cm^2^, as determined by RT-qPCR with 16S rRNA as the reference gene, from three independent biological replicates. Statistical significance was determined by one-way ANOVA (*p < 0.05; ***p < 0.001). (C) Hierarchical clustering heatmap of key DEG clusters distinguishing the control and aBL (420 nm)-treated groups. (D) Principal component analysis (PCA) of transcriptomic profiles, showing clear separation between control and 420 nm aBL-treated groups along PC1 (75.3% of variance). (E) KEGG enrichment bubble plot (GeneRatio) showing the top enriched pathways among DEGs. (F) Gene Ontology (GO) enrichment bubble plot (GeneRatio) showing the top enriched biological process, cellular component, and molecular function terms among DEGs. Transcriptomic sequencing was performed on the Illumina NovaSeq platform. (For interpretation of the references to color in this figure legend, the reader is referred to the web version of this article.)

Autolysis is responsible for biofilm formation under diverse circumstances (Yang et al., 2024). To understand its role in the aBL-mediated inhibition of L. *monocytogenes*, the autolysis extent was then examined upon blue light illumination. As shown in Fig. 4D, the autolysis ratio in the 420 nm group was significantly elevated compared to the intact group at 12 h (*p* < 0.01), 24 h (*p* < 0.05), and 36 h (*p* < 0.01), indicating that aBL promotes bacterial autolysis during both the attachment and maturation phases of biofilm development. By contrast, the 440 nm group showed a moderate and less consistent increase in autolysis. From transcriptomic data, we detected the upregulation of *recA* (Fig. 5B), a gene shown responsible for the LexA-autolysis promotion in *Myxococcus xanthus* (Sheng et al., 2020). Despite this correlation has not yet established in L. *monocytogenes*, it is plausible to hypothesize that the upregulated *recA* might, at least partly, account for the stimulated autolysis in the condition of blue light illumination. These findings suggest that aBL disrupts biofilm architecture not only by inhibiting initial colonization but also by accelerating autolytic processes during biofilm maturation.

### 3.4 Transcriptomic analysis reveals the molecular mechanisms underlying aBL-mediated antibacterial activity

At the molecular level, whole-transcriptome sequencing of L. *monocytogenes* exposed to 420 nm aBL at 108 J/cm^2^ was performed using the Illumina NovaSeq platform. Of the 2749 genes detected overall, 68 were unique to the control group, 57 were unique to the aBL-treated group, and 2624 were shared between the two conditions (Fig. S4A); this large shared fraction reflects stable expression of essential housekeeping genes under both conditions. Focusing on differentially expressed genes (DEGs) defined by the stricter criteria of |log₂FC| > 1 and padj <0.05, we identified 646 significantly altered genes, visualized in the volcano plot (Fig. 5A), indicating that blue light exposure substantially reshaped gene expression in L. *monocytogenes*. Notably, key antioxidant stress response and DNA repair genes — including *kat*, and *recA* — were among the most prominently upregulated genes (e.g., *kat*: log₂FoldChange = 1.719, padj <0.001), which were further validated by RT-qPCR (Fig. 5B). Hierarchical clustering of the DEGs (Fig. 5C) clearly separated the aBL-treated samples from the controls and highlighted co-regulated gene clusters, including the upregulated stress-response genes, and principal component analysis (Fig. 5D) confirmed a clear, reproducible separation between the two groups (PC1 = 75.3% of variance). Of particular interest, a gene interaction network analysis of porphyrin- and cobalamin-related biosynthesis genes (*cbi* and *hem* gene families) and a connected folate/amino-acid biosynthesis module (*folC*, *folD*, *trpA*, *trpD*, *trpF*, *ilvA*, *serS*) revealed coordinated upregulation of both modules following aBL treatment (Fig. S4C). This pattern suggests that aBL-induced photo-oxidative stress triggers a compensatory transcriptional response that replenishes porphyrin precursors.

In terms of pathways, KEGG enrichment analysis showed that DEGs were significantly enriched in multiple metabolic pathways, most prominently biosynthesis of secondary metabolites, biosynthesis of amino acids, and branched-chain amino acid (valine, leucine, and isoleucine) biosynthesis, alongside ABC transporters and carbon/porphyrin metabolism (Fig. 5E; Fig. S4B). Complementing this, GO enrichment analysis (Fig. 5F) showed that DEGs were predominantly associated with translation and ribosome-related terms, transport processes, and metal ion binding. Together, these KEGG and GO results are consistent with aBL-induced perturbation of membrane transport, redox and metal-ion homeostasis, and translational machinery, corroborating the activation of antioxidant stress and DNA repair pathways.

### 3.5 aBL effectively reduces L. *monocytogenes* contamination in orange juice while preserving quality

**Fig. 6:**
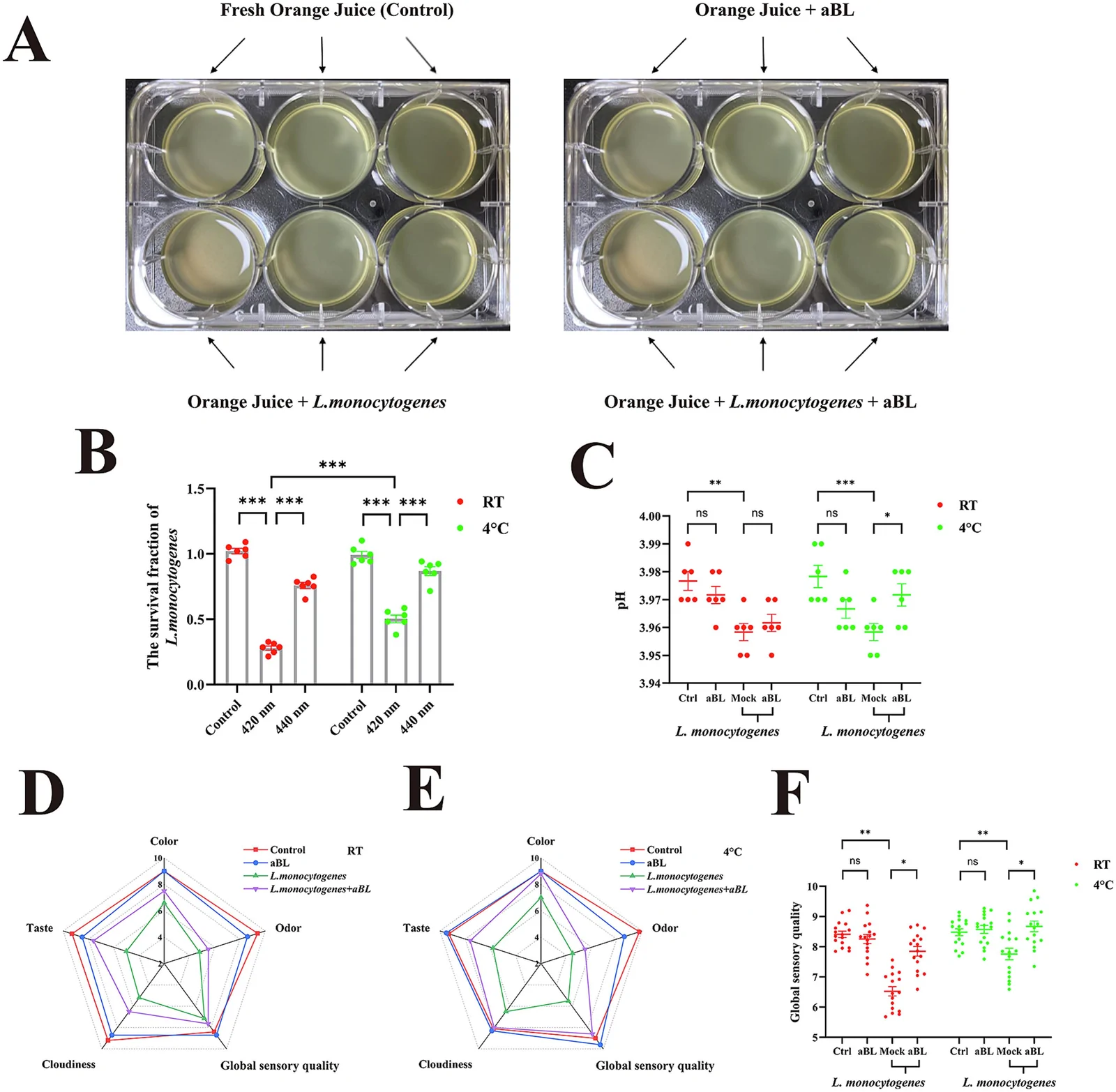
aBL decontaminates fresh orange juice from *Listeria monocytogenes* while preserving sensory quality. (A) Photographs of 6-well plates showing fresh orange juice under four treatment conditions: fresh orange juice (control), orange juice treated with aBL, orange juice inoculated with L. *monocytogenes* (Mock), and orange juice inoculated with L. *monocytogenes* and treated with aBL, stored for 48 h at room temperature (RT) or 4 °C. (B) Survival fraction of L. *monocytogenes* in orange juice following treatment with 420 nm or 440 nm aBL at 108 J/cm^2^ under RT (red) or 4 °C (green) conditions. (C) pH of orange juice under the indicated treatment conditions at RT and 4 °C. “Mock” refers to L. *monocytogenes*-inoculated juice without aBL treatment, and “aBL” under the L. *monocytogenes* group refers to inoculated juice subjected to aBL treatment. Additional physicochemical quality parameters, such as polyphenol oxidase (PPO) relative activity, soluble solids content (°Brix), and total polyphenol content, are shown in Fig. S5. (D, E) Radar charts showing sensory evaluation scores for color, odor, global sensory quality, cloudiness, and taste of orange juice under the four treatment conditions at RT (D) and 4 °C (E), assessed by 16 trained evaluators using a 10-point hedonic scale (1 = poor, 10 = excellent; minimum acceptable score = 5), with untreated orange juice serving as the reference. (F) Global sensory quality of orange juice under the indicated treatment conditions at RT and 4 °C, assessed by the trained panel described above; consumer overall acceptability scores from a separate panel of 25 untrained consumers are shown in Fig. S5D. Data for panels (B, C) are presented as mean ± SEM from three independent experiments; sensory scores in (D—F) reflect the trained panel described above. Statistical significance was determined by one-way ANOVA (ns, not significant; *p < 0.05; **p < 0.01; ***p < 0.001). (For interpretation of the references to color in this figure legend, the reader is referred to the web version of this article.)

It is of practical importance to test the adaptability of aBL in various scenarios of food storage. In terms of liquid food decontamination, fresh orange juice artificially inoculated with L. *monocytogenes* was subjected to aBL treatment at 108 J/cm^2^ and stored for 48 h under both room temperature (RT) and refrigeration conditions (Fig. 6A). As shown in Fig. 6B, both 420 nm and 440 nm aBL significantly weakened the survival of L. *monocytogenes* in orange juice under both storage temperatures compared to untreated controls (*p* < 0.001). Notably, 420 nm aBL consistently achieved greater bactericidal efficacy than 440 nm at both temperatures, with more pronounced reductions observed at RT. Specifically, at RT, 420 nm reduced bacterial counts by 2.1 log₁₀ CFU/mL, whereas 440 nm achieved only a 0.9 log₁₀ reduction, demonstrating that 420 nm was approximately 2.3-fold more effective in this liquid food matrix. These results confirm that aBL is effective in reducing listeria contamination in a liquid food matrix under realistic storage conditions.

To assess whether aBL treatment compromises the quality of orange juice, multiple physicochemical and sensory parameters were evaluated. pH measurements showed no significant differences at 4 °C, while minor but statistically significant differences were observed at RT pending light exposure (Fig. 6C). Polyphenol oxidase (PPO) relative activity was significantly reduced in the aBL-treated groups at RT (*p* < 0.001), suggesting that aBL may yield unexpected benign influence by partially inhibiting enzymatic browning (Fig. S5A). Soluble solids content (°Brix) was marginally elevated in the 420 nm aBL group at RT (*p* < 0.001), albeit of no similar variances detected at 4 °C (Fig. S5B). Total polyphenol content showed no significant changes across treatment groups under either storage condition (Fig. S5C). Sensory evaluation by a panel of 16 trained evaluators, supplemented by a separate 25-person untrained consumer acceptance test, using a 10-point hedonic scale revealed no meaningful organoleptic differences attributable to aBL treatment under alternative storage conditions, as illustrated by overlapping radar charts for color, odor, cloudiness, taste, and global sensory quality (Fig. 6D, E). Consistently, the global sensory quality scores did not differ significantly due to the introduction of aBL (Fig. 6F). This conclusion was corroborated by a separate panel of 25 untrained consumers, whose overall acceptability ratings followed the same pattern, with no significant difference between aBL-treated and untreated juice (Fig. S5D). Triangle discrimination testing (Table S2) indicates that aBL treatment introduces no perceptible sensory change. This consistent overlap across all attributes provides robust evidence that aBL does not cause sensory deterioration in orange juice. In summary, aBL effectively reduces L. *monocytogenes* contamination in orange juice while preserving its essential physicochemical and sensory qualities.

### 3.6 aBL decontaminates sliced and packaged surimi products while maintaining food quality

**Fig. 7:**
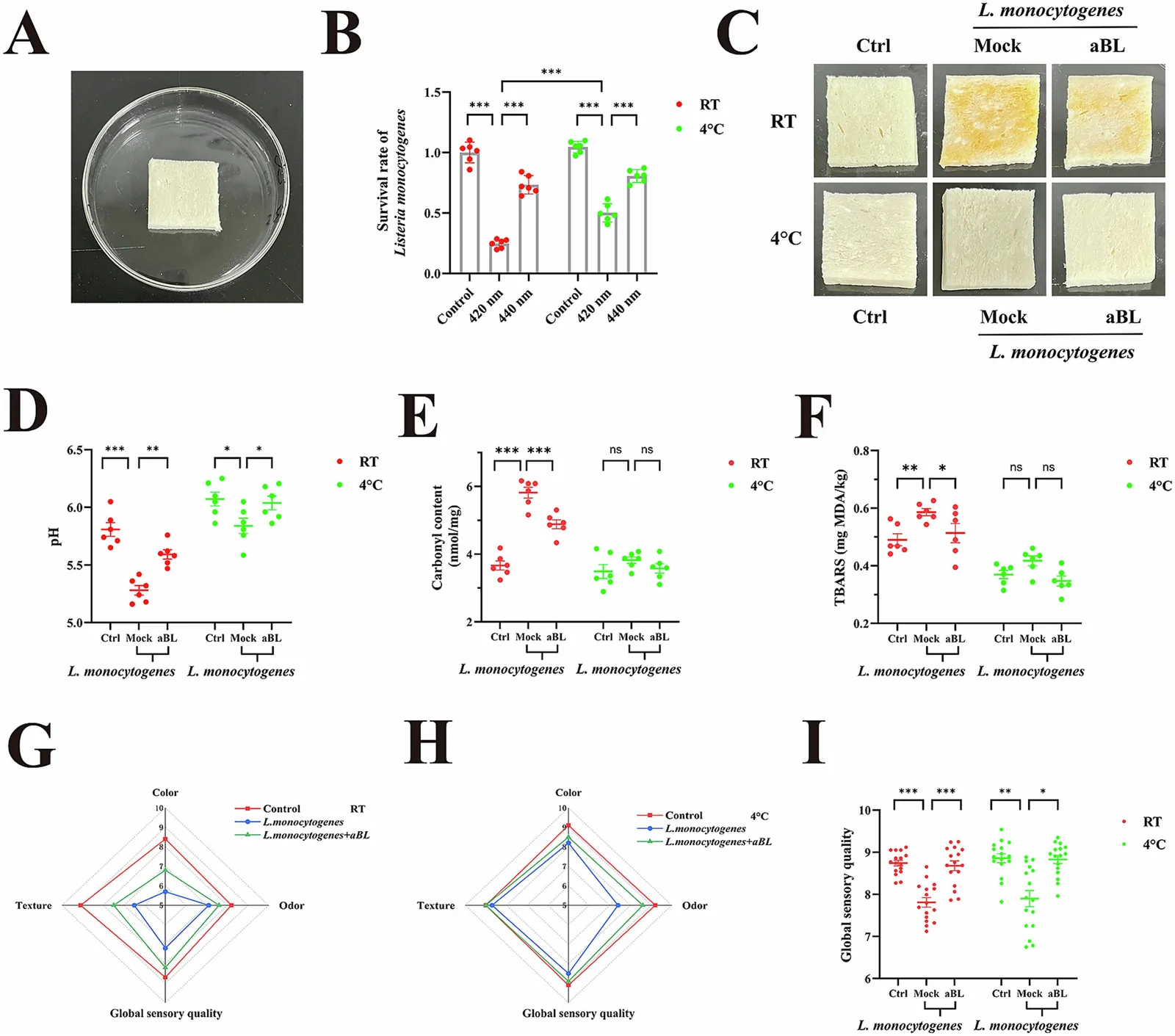
aBL decontaminates the sliced surimi products from *Listeria monocytogenes* while preserving sensory quality. (A) Photograph of a representative sliced surimi product used in this study. (B) Survival rate of L. *monocytogenes* on sliced surimi following treatment with 420 nm or 440 nm aBL at 108 J/cm^2^ under room temperature (RT, red) or 4 °C (green) conditions. (C) Photographs showing the appearance of sliced surimi under three treatment conditions — untreated control (Ctrl), *L. monocytogenes*-inoculated without aBL treatment (Mock), and L. *monocytogenes*-inoculated with aBL treatment (aBL) — stored for 48 h at RT or 4 °C. (D—F) Chemical quality parameters of sliced surimi under the indicated treatment conditions at RT and 4 °C, including pH (D), carbonyl content (nmol/mg) (E), and thiobarbituric acid reactive substances (TBARS, mg MDA/kg) (F). Textural properties (chewiness, cohesiveness, and hardness) are shown in Fig. S6. (G, H) Radar charts showing sensory evaluation scores for color, odor, texture, and global sensory quality of sliced surimi under the three treatment conditions at RT (G) and 4 °C (H), assessed by 16 trained evaluators using a 10-point hedonic scale (1 = poor, 10 = excellent; minimum acceptable score = 5), with untreated surimi slices serving as the reference. (I) Global sensory quality of sliced surimi under the indicated treatment conditions at RT and 4 °C, assessed by the trained panel described above; consumer overall acceptability scores from the separate 25-person consumer panel are shown in Fig. S6D. Data for panels (B, D—F) are presented as mean ± SEM from three independent experiments; sensory scores in (G–I) reflect the trained panel described above. Statistical significance was determined by one-way ANOVA (ns, not significant; *p < 0.05; **p < 0.01; ***p < 0.001). (For interpretation of the references to color in this figure legend, the reader is referred to the web version of this article.)

Surimi products are easily contaminated by L. *monocytogenes* during storage (Yamazaki et al., 2003). To further evaluate the applicability of aBL to solid food products, sliced surimi was used as a model system. Sliced surimi was inoculated with L. *monocytogenes* and then exposed to blue light at RT or refrigeration (Fig. 7A). It was found that aBL pronouncedly decreased L. *monocytogenes* counts on the surimi surface under both temperatures (*p* < 0.001), with 420 nm again exhibiting superior bactericidal efficacy (Fig. 7B). Quantitatively, at RT, 420 nm reduced surface counts by 2.5 log₁₀ CFU/g, compared to 1.1 log₁₀ CFU/g for 440 nm, confirming the wavelength-dependent propensity observed in liquid matrices. Visual inspection revealed that aBL-treated surimi maintained a comparable appearance to untreated controls, while Mock-inoculated samples showed visible discoloration, particularly at RT (Fig. 7C, Table 1), which implies the co-existence of spoilage microbes with L. *monocytogenes*.

**Table 1:** Color parameters of surimi products under different processing conditions.

Color parameter		Sample	Treatment	
			RT	4°C
ΔE*ab (Dimensionless)	L*	Control	51.29 ± 2.02^a^	64.75 ± 2.74^a^
		*L. monocytogenes*	48.47 ± 2.55^a^	**57.96 ± 0.60**^**b**^
		*L. monocytogenes* + aBL	47.80 ± 2.42^a^	**60.69 ± 4.96**^**b**^
	a*	Control	- 0.59 ± 0.12^c^	- 1.08 ± 0.37^a^
		*L. monocytogenes*	**7.22 ± 0.40**^**a**^	- 0.81 ± 0.12^a^
		*L. monocytogenes* + aBL	**1.25 ± 0.41**^**b**^	- 0.91 ± 0.15^a^
	b*	Control	12.00 ± 0.26^c^	9.75 ± 0.84^b^
		*L. monocytogenes*	**24.80 ± 0.86**^**a**^	10.06 ± 0.16^b^
		*L. monocytogenes* + aBL	**16.01 ± 0.86**^**b**^	**11.05 ± 0.45**^**a**^

L*, Lightness; a*, Redness-Greenness; b*, Yellowness-Blueness. Different upper letters indicate statistically significant differences (*p* < 0.05) based on multiple-comparison tests, while identical letters denote no significant difference. (Bold indicates *p* < 0.05 versus the control group).

Assessment of chemical quality parameters showed that pH values were lower in the inoculation group at RT (*p* < 0.05), indicating that bacterial contamination accelerated acidification, which was further mitigated by aBL treatment (Fig. 7D). Carbonyl content, a marker of protein oxidation, was elevated upon listeria inoculum at RT (*p* < 0.001), and aBL treatment restored it to levels comparable to the untreated slice (Fig. 7E). TBARS value, indicative of lipid oxidation, was also resumed by aBL treatment, while no fluctuations were observed under refrigerated storage (Fig. 7F). Critically, the lack of increased TBARS in aBL-treated samples strongly indicates that aBL does not induce lipid photo-oxidation at the applied doses, which is a key safety consideration for real-world applications. Regarding textural properties, chewiness, cohesiveness, and hardness were all compromised by contamination, whereas aBL-treated surimi exhibited comparable textural properties with the untreated product (Fig. S6, Table 2). The apparent improvement in textural parameters (chewiness and hardness) upon aBL treatment is likely not due to direct protein cross-linking induced by blue light, but rather attributable to the effective elimination of pathogenic and spoilage microorganisms, thereby suppressing proteolytic and lipolytic degradation of the myofibrillar protein matrix during subsequent storage. Sensory evaluation confirmed these findings, with aBL-treated slice maintaining scores for color, odor, texture, and global sensory quality (Fig. 7G-I). Consumer overall acceptability ratings from the separate 25-person consumer panel followed the same pattern (Fig. S6D). Triangle discrimination testing (Table S3) showed that Mock-inoculated surimi was reliably distinguished from the untreated control (13–15 of 16 correct identifications), whereas aBL-treated surimi was not (7–8 of 16 correct identifications), corroborating the absence of a perceptible sensory change.

**Table 2:** Texture of surimi products under different processing conditions.

Sample parameters		Sample	Treatment	
			RT	4°C
Texture	Hardness (g)	Control	927.67 ± 113.74^a^	1627.17 ± 205.74^a^
		*L. monocytogenes*	**267.19 ± 149.18**^**c**^	1435.77 ± 261.13^a^
		*L. monocytogenes* + aBL	**411.85 ± 101.12**^**b**^	1499.06 ± 320.78^a^
	Adhesiveness (g.*sec*)	Control	- 7.15 ± 3.29^a^	- 6.94 ± 1.97^a^
		*L. monocytogenes*	**- 29.39 ± 15.49**^**b**^	- 6.96 ± 2.00^a^
		*L. monocytogenes* + aBL	**- 38.80 ± 12.79**^**b**^	- 7.94 ± 1.95^a^
	Springiness (%)	Control	0.36 ± 0.07^a^	0.80 ± 0.08^a^
		*L. monocytogenes*	0.30 ± 0.06^a^	0.77 ± 0.17^a^
		*L. monocytogenes* + aBL	0.40 ± 0.15^a^	**0.62 ± 0.06**^**b**^
	Cohesiveness (g)	Control	0.86 ± 0.07^a^	0.86 ± 0.10^a^
		*L. monocytogenes*	**0.44 ± 0.06**^**c**^	**0.72 ± 0.09**^**b**^
		*L. monocytogenes* + aBL	**0.66 ± 0.20**^**b**^	0.79 ± 0.13^ab^
	Gumminess (Dimensionless)	Control	788.28 ± 57.74^a^	1158.08 ± 111.17^a^
		*L. monocytogenes*	**158.12 ± 31.78**^**b**^	1151.21 ± 174.70^a^
		*L. monocytogenes* + aBL	**179.87 ± 20.26**^**b**^	1024.40 ± 192.76^a^
	Chewiness (Dimensionless)	Control	321.20 ± 24.79^a^	722.25 ± 78.43^a^
		*L. monocytogenes*	**48.38 ± 17.32**^**c**^	**589.96 ± 51.39**^**b**^
		*L. monocytogenes* + aBL	**105.79 ± 10.55**^**b**^	645.83 ± 66.84^ab^
	Resilience (%)	Control	0.38 ± 0.05^a^	0.42 ± 0.07^a^
		*L. monocytogenes*	**0.20 ± 0.07**^**b**^	**0.21 ± 0.04**^**b**^
		*L. monocytogenes* + aBL	**0.14 ± 0.02**^**c**^	**0.23 ± 0.05**^**b**^
	Overall quality score (Dimensionless)	Control	0.58 ± 0.03^a^	1.00 ± 0.02^a^
		*L. monocytogenes*	**0.04 ±** **0.04**^**b**^	**0.80 ± 0.05**^**b**^
		*L. monocytogenes* + aBL	**0.15 ± 0.03**^**c**^	**0.79 ± 0.04**^**b**^

Different letters indicate statistically significant differences (*p* < 0.05) based on multiple-comparison tests, while identical letters denote no significant difference.

(Bold indicates *p* < 0.05 versus the control group)

Overall Score j  = $\sum_{i=1}^{7}\mathrm{Wi}\times \mathrm{Xi}\;$, and the individual weight is assigned according to actual edible characteristics of surimi, and listed as follows: Hardness, 0.20; Adhesiveness, 0.05; Springiness, 0.15; Cohesiveness, 0.15; Gumminess, 0.15; Chewiness, 0.20; Resilience, 0.10.

**Fig. 8:**
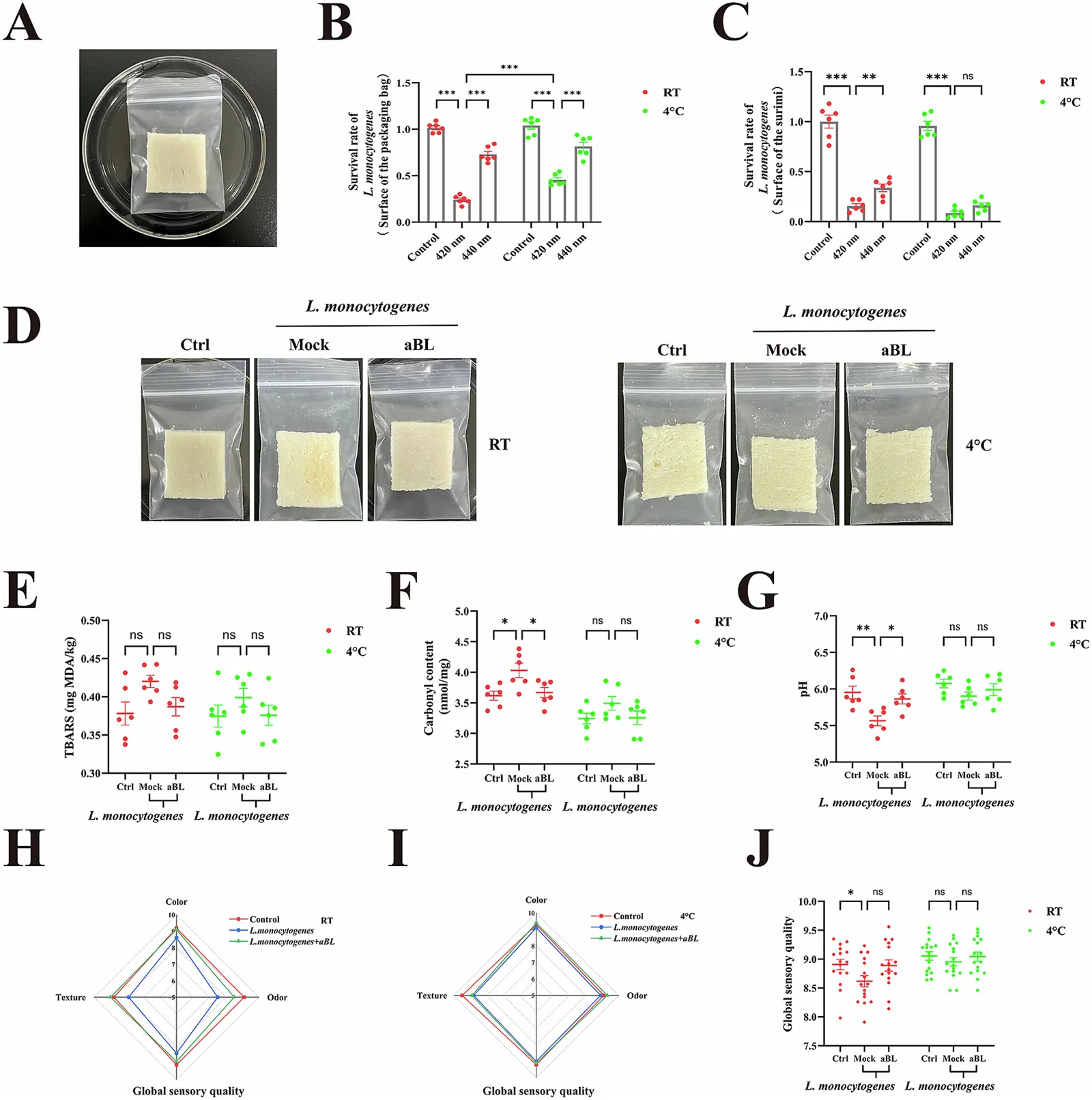
aBL decontaminates the packaged surimi products from *Listeria monocytogenes* while preserving sensory quality. (A) Photograph of a representative packaged sliced surimi product used in this study. (B) Survival rate of L. *monocytogenes* on the surface of the packaging bag following treatment with 420 nm or 440 nm aBL at 108 J/cm^2^ under room temperature (RT, red) or 4 °C (green) conditions. (C) Survival rate of L. *monocytogenes* inside the packaged surimi following treatment with 420 nm or 440 nm aBL at 108 J/cm^2^ under RT or 4 °C conditions. (D) Photographs showing the appearance of packaged sliced surimi under three treatment conditions stored for 48 h at RT or 4 °C. (E–G) Chemical quality parameters of packaged sliced surimi under the indicated treatment conditions at RT and 4 °C, including thiobarbituric acid reactive substances (TBARS, mg MDA/kg) (E), carbonyl content (nmol/mg) (F), and pH (G). Textural properties (chewiness, cohesiveness, and hardness) are shown in Fig. S7. (H, I) Radar charts showing sensory evaluation scores for color, odor, texture, and global sensory quality of packaged sliced surimi under the three treatment conditions at RT (H) and 4 °C (I), assessed by 16 trained evaluators using a 10-point hedonic scale. (J) Global sensory quality of packaged surimi under the indicated treatment conditions at RT and 4 °C, assessed by the trained panel described above; consumer overall acceptability scores from the separate 25-person consumer panel are shown in Fig. S7D. Data for panels (B, C, E–G) are presented as mean ± SEM from three independent experiments; sensory scores in (H–J) reflect the trained panel described above. Statistical significance was determined by one-way ANOVA (ns, not significant; *p < 0.05; **p < 0.01; ***p < 0.001). (For interpretation of the references to color in this figure legend, the reader is referred to the web version of this article.)

We further investigated whether aBL could facilitate the decontamination of packaged surimi products — a scenario more representative of practical food storage (Fig. 8A). The 108 J/cm^2^ illumination of blue light significantly removed L. *monocytogenes* from the surface of the packaging bag under both temperatures (*p* < 0.001), with 420 nm showing greater potency than 440 nm (Fig. 8B). It's noteworthy that bacterial counts inside the packaged surimi were also drastically curbed following aBL treatment, particularly at RT with 420 nm and 440 nm (*p* < 0.001 and *p* < 0.01, respectively), and 420 nm at 4 °C (*p* < 0.001) (Fig. 8C). This result demonstrates that aBL can penetrate transparent packaging to exert bactericidal effects on the enclosed surimi product. Specifically, on the packaging surface at RT, 420 nm achieved a 3.5 log₁₀ reduction compared to 2.0 log₁₀ for 440 nm, further highlighting the superior practical utility of the shorter wavelength.

Visual and colorimetric analyses did not detect apparent color alterations owing to aBL treatment (Fig. 8D, Table 3). Quality assessment of packaged surimi showed that TBARS values did not differ across treatment groups (Fig. 8E), which again confirms the absence of lipid photo-oxidation and reinforces the safety profile of aBL even in packaged systems, indicating that aBL does not exacerbate lipid oxidation through the packaging. The inoculum-driven anomaly of carbonyl content and pH value was partially attenuated by light exposure (Fig. 8F, G). Textural analysis revealed that chewiness and hardness were also maintained or improved by aBL treatment (Fig. S7, Table 4). Similar to unpackaged surimi, sensory evaluation showed that aBL treatment did not significantly alter the color, odor, texture, or global sensory quality of packaged surimi, as demonstrated by the overlapping radar charts and global sensory quality scores (Fig. 8H-J); consumer overall acceptability ratings from the separate 25-person panel followed the same pattern (Fig. S7D). Triangle discrimination testing (Table S4) confirmed that Mock-inoculated packaged surimi was reliably distinguished from the untreated control (12–14 of 16 correct identifications), whereas aBL-treated packaged surimi was not (5–7 of 16 correct identifications), further supporting that aBL treatment does not introduce a perceptible sensory change.

**Table 3:** Color parameters of surimi products (placed in packaging bags) under different processing conditions.

Color parameter		Sample	Treatment	
			RT	4°C
ΔE*ab (Dimensionless)	L*	Control	49.22 ± 1.50^b^	66.22 ± 0.60^a^
		*L. monocytogenes*	49.91 ± 3.14^b^	65.61 ± 1.35^a^
		*L. monocytogenes* + aBL	**52.59 ± 0.88**^**a**^	**62.28 ± 1.85**^**b**^
	a*	Control	- 1.39 ± 0.21^b^	- 1.06 ± 0.13^b^
		*L. monocytogenes*	**3.12 ± 0.45**^**a**^	**- 0.60 ± 0.26**^**a**^
		*L. monocytogenes* + aBL	- 1.32 ± 0.10^b^	**- 1.30 ± 0.05**^**c**^
	b*	Control	10.04 ± 0.50^b^	10.82 ± 1.15^a^
		*L. monocytogenes*	**15.44 ± 0.60**^**a**^	10.93 ± 1.68^a^
		*L. monocytogenes* + aBL	10.66 ± 0.11^b^	11.04 ± 1.29^a^

L*, Lightness; a*, Redness-Greenness; b*, Yellowness-Blueness. Different upper letters indicate statistically significant differences (*p* < 0.05) based on multiple-comparison tests, while identical letters denote no significant difference. (Bold indicates *p* < 0.05 versus the control group).

**Table 4:** Texture of surimi products (placed in packaging bags) under different processing conditions.

Sample parameters		Sample	Treatment	
			RT	4°C
Texture	Hardness (g)	Control	966.25 ± 110.35^a^	1303.17 ± 152.32^a^
		*L. monocytogenes*	**576.40 ± 59.35**^**b**^	1225.42 ± 142.33^a^
		*L. monocytogenes* + aBL	903.52 ± 87.84^a^	1240.62 ± 201.53^a^
	Adhesiveness (g.sec)	Control	- 9.47 ± 3.29^a^	- 6.21 ± 2.33^a^
		*L. monocytogenes*	**- 15.96 ± 7.03**^**b**^	- 6.56 ± 1.36^a^
		*L. monocytogenes* + aBL	- 9.20 ± 4.89^a^	- 8.20 ± 3.46^a^
	Springiness (%)	Control	0.45 ± 0.07^a^	0.76 ± 0.05^a^
		*L. monocytogenes*	0.39 ± 0.16^a^	**0.69 ± 0.09**^**b**^
		*L. monocytogenes* + aBL	0.44 ± 0.10^a^	0.71 ± 0.04^ab^
	Cohesiveness (g)	Control	0.91 ± 0.18^a^	0.88 ± 0.19^a^
		*L. monocytogenes*	**0.76 ± 0.15**^**b**^	0.81 ± 0.05^a^
		*L. monocytogenes* + aBL	0.81 ± 0.09^ab^	0.86 ± 0.11^a^
	Gumminess (Dimensionless)	Control	850.63 ± 37.74^a^	1261.59 ± 123.58^a^
		*L. monocytogenes*	**681.70 ± 41.78**^**b**^	1189.63 ± 192.77^a^
		*L. monocytogenes* + aBL	**766.52 ± 40.26**^**c**^	1169.50 ± 147.31^a^
	Chewiness (Dimensionless)	Control	389.23 ± 14.78^a^	794.57 ± 43.28^a^
		*L. monocytogenes*	**261.62 ± 8.32**^**b**^	**716.21 ± 36.96**^**b**^
		*L. monocytogenes* + aBL	**332.11 ± 9.55**^**c**^	**705.46 ± 51.83**^**b**^
	Resilience (%)	Control	0.40 ± 0.08^a^	0.43 ± 0.02^a^
		*L. monocytogenes*	**0.33 ± 0.03**^**b**^	0.39 ± 0.06^a^
		*L. monocytogenes* + aBL	0.35 ± 0.12^ab^	0.43 ± 0.10^a^
	Overall quality score (Dimensionless)	Control	0.86 ± 0.03^a^	0.96 ± 0.25^a^
		*L. monocytogenes*	**0.26 ± 0.63**^**b**^	**0.15 ± 0.31**^**b**^
		*L. monocytogenes* + aBL	**0.61 ± 0.16**^**c**^	**0.30 ± 0.03**^**b**^

Different letters indicate statistically significant differences (*p* < 0.05) based on multiple-comparison tests, while identical letters denote no significant difference.

(Bold indicates *p* < 0.05 versus the control group)

Overall Score j  = $\sum_{i=1}^{7}\mathrm{Wi}\times \mathrm{Xi}\;$, and the individual weight is assigned according to actual edible characteristics of surimi, and listed as follows: Hardness, 0.20; Adhesiveness, 0.05; Springiness, 0.15; Cohesiveness, 0.15; Gumminess, 0.15; Chewiness, 0.20; Resilience, 0.10.

Taken together, aBL is fully compatible with both unpackaged and packaged surimi storage, effectively reducing L. *monocytogenes* contamination while maintaining their textural integrity, chemical stability, and sensory characteristics.

## 4 Discussion

In this study, a systematic characterization of aBL as a decontamination strategy against *Listeria monocytogenes* was conducted amid multiple food matrices. Based on our findings, the bactericidal mechanisms of aBL were interrogated at both cellular and molecular levels in a comprehensive manner, with respect to both planktonic and biofilms. Moreover, aBL was validated as an effective and quality-preserving decontamination approach in liquid, solid, and packaged food formats, including under refrigerated conditions - the exact circumstances under which L. *monocytogenes* poses severe threat to food safety. The discovery here provides a scientifically grounded basis for the broader adoption of aBL in food preservation.

As a Gram-positive organism, *L. monocytogenes* is theoretically well-suited to photodynamic inactivation (Kasimova et al., 2014), which is demonstrated by the current finding that aBL exerts profoundly detrimental effect on L. *monocytogenes* (Fig. 1, Fig. 2). This is consistent with our PI uptake and leakage assays, which show membrane compromise within minutes. This cascade explains why aBL is so effective even against stationary-phase cells, which have reduced repair activity. Up to 3–4 log reductions in L. *monocytogenes* viability at 108–144 J/cm^2^ is broadly in line with, yet variable relative to, the existing literature. Josewin et al. (2018) stated approximately 2–3 log reductions on cantaloupe rinds using 405 nm light, while Olszewska et al. (2023) documented up to 5 log reductions in dried listeria cells at comparable wavelengths with prolonged exposure. Rivera-Santiago and Diez-Gonzalez (2025) similarly emphasized that dose and surface material are critical determinants of killing efficiency. The variability might reflect differences in experimental conditions, including the initial bacterial load, cell physiological state, and the specific strain under study, all of which are recognized as determinants of aBL efficacy (Hur & Diez-Gonzalez, 2025). Still, consistent with prior studies, 420 nm aBL exhibited superior efficacy relative to longer wavelengths within the blue spectrum (Xiao & Zhang, 2025). This wavelength dependence is primarily explained by the known absorption spectrum of bacterial porphyrins, which typically peaks in the 405–420 nm (Soret band) region and declines toward 440–460 nm; because photon absorption is rate-limiting for ROS generation in photodynamic inactivation, wavelengths closer to this absorption maximum are expected to generate more ROS per unit dose, though direct photochemical confirmation would be needed to formally establish this. A second, complementary factor may be photon intensity: for a given driving current, shorter-wavelength LEDs can deliver a higher photon flux than longer-wavelength LEDs of similar design, which would act in the same direction as the absorption effect described above, consistently favoring 420 nm over longer wavelengths in our data. The similar supremacy of lower band of wavelength is also detected in its applications in other foodborne pathogens (Wang et al., 2024).

Building on this general susceptibility, several aspects of the ROS-mediated killing cascade merit nuanced mechanistic consideration. Given that aBL acts via photoexcitation of endogenous porphyrins, the primary ROS species generated are expected to include singlet oxygen (^1^O₂) via the Type II photodynamic pathway and superoxide anion (O₂•^−^)/hydrogen peroxide (H₂O₂) via Type I electron-transfer reactions. Rather than being concentrated at the cytoplasmic membrane, fluorescence lifetime imaging of endogenous porphyrins indicates that these chromophores are distributed relatively homogeneously throughout the cell, reflecting the general lack of subcellular compartmentalization in bacteria (Leanse et al., 2023). ROS generation is therefore likely to occur throughout the cell rather than being confined to the membrane, though the short diffusion radius of ROS such as singlet oxygen means that damage remains concentrated near each site of generation - plausibly affecting the membrane, cytoplasmic proteins, and nucleic acids over a similar timeframe rather than in a strict sequence. Consistent with an induced antioxidant response, our transcriptomic data show upregulation of *kat* (catalase) and *spxA*, indicating activation of oxidative-stress defenses, while the concurrent upregulation of *recA* points to activation of the SOS DNA-repair response. Our phenotypic assays captured clear membrane disruption and leakage of intracellular DNA and protein within the treatment window (Fig. 3), whereas the DNA-repair transcriptional response was only assessed at a single endpoint; our data therefore cannot establish whether membrane damage precedes DNA damage or whether both occur in parallel, and resolving this would require time-resolved sub-cellular imaging, which we identify as a direction for future work.

The responsiveness of L. *monocytogenes* to blue light is assumably mediated by the stress sigma factor σB (SigB) and blue light sensor Lmo0799 (Dorey et al., 2019; O'Donoghue et al., 2016), stressing the importance of gene expression regulation. This notion is further supported by the current pathway analyses (Fig. 5E, F). In addition, we also discovered the upregulation of *spxA*, another global transcriptional regulator (Shi et al., 2021), in response to blue light illumination, which offers the growing evidence that a complex regulatory network underlies the bacterial responsiveness toward aBL rather than a predominant role of SigB. In parallel, the expressional alteration of *kat*, encoding catalase, indicates the activation of antioxidant defense programs in an attempt to neutralize aBL-generated ROS, constituting an integral part of listeria responsiveness against antioxidative stress, together with the induction of *recA*, a central effector of the DNA damage response. The transcriptional reprogramming also exhibits consistency with subcellular changes such as ROS stimulation and DNA damage, forming a uniform mechanistic signaling. Of note, gene expression changes vary relying on the wavelength used (420 nm vs. 440 nm), further substantiating that the molecular stress response of L. *monocytogenes* is tuned to the wavelength of irradiation. This might reflect a type of wavelength-specific regulation, which was previously proposed in *E. coli* (Khan et al., 2023).

The efficacy of aBL spans three distinct sets of food application contexts, underscoring its versatility as a decontamination tool. Of particular note, aBL is able to trim L. *monocytogenes* counts not only on the outer surface of packaging bags but also within the packaged surimi per se. This observation might suggest the penetration of aBL of food-grade polyethylene (PE) packaging to exert bactericidal effects on the enclosed product. PE packaging is among the most widely used materials in the seafood industry, valued for its flexibility, mechanical strength (Gerassimidou et al., 2023). The compatibility with aBL usage is practically appealing because it would, in principle, allow aBL to be applied post-packaging without disrupting the integrity of the package or the established production workflow. However, the extent of through-package decontamination likely depends on packaging material thickness, optical clarity, and geometry of light delivery, etc. (Otter et al., 2013), parameters that were not systematically investigated in this study. Whether similar efficacy would be achievable with alternative packaging materials remains an open question, warranting future work to address.

A prominent observation in all food experiments is that aBL treatment did not compromise food quality and, in several instances, unexpectedly improved some textual or sensory attributes (Fig. 6, Fig. 7, Fig. 8). In orange juice, aBL-treated samples exhibited reduced PPO activity, which could be attributable to the photo-oxidative inactivation of this copper-containing enzyme by blue light. The light-mediated changes are assumed to attenuate enzymatic browning, although this mechanistic interpretation requires direct experimental validation. In surimi, aBL-treated slices displayed superior chewiness, cohesiveness, and hardness compared to non-irradiation group. This desirable alteration likely resulted from the aBL-mediated inhibition of spoilage microorganisms beyond L. *monocytogenes*. Actually, it is long debated that aBL could yield the relatively broad-spectrum bactericidal activity against a collection of foodborne pathogens and spoilage organisms (Hur & Diez-Gonzalez, 2025; Leanse et al., 2021). This extra merit also has alternative explanations – sustained blue light irradiation may exert direct effects on protein structure, enzymatic activity or cross-linking within the surimi matrix in ways that alter textural properties. This argument is supported by a prior study that blue light can mediate signal transduction by finetuning the structure of a bacteria BLUF photoreceptor (Jung et al., 2005). Nonetheless, it is noteworthy that both proposed mechanisms remain complete, and further studies are needed to resolve the relevant concerns.

Our observation that 420 nm aBL significantly reduces biofilm biomass at 12 h (early attachment) and 36 h (maturation) but less so at 24 h warrants attention. Theoretically, at 12 h, cells are actively adhering and producing EPS (Akter et al., 2025); they are metabolically active and exposed to light, making them highly susceptible to ROS-induced damage. At 36 h, the biofilm is thick, yet 420 nm still reduces biomass - likely due to its higher photon energy. The reduced effect at 24 h may reflect a transitional phase where EPS production outpaces oxidative damage. Additionally, the enhanced autolysis we observed at 12 and 36 h suggests that aBL accelerates programmed cell death, which contributes to biofilm disruption at both stages - by inhibiting colonization in early phases and by disaggregating mature clusters in later phases. This dual mechanism makes aBL particularly promising for biofilm control in food processing environments.

We observed consistently higher bactericidal efficacy at room temperature (RT) than at 4 °C across all food matrices. This temperature dependence can be attributed to several factors: (i) higher metabolic activity at RT leads to faster respiration and potentially higher endogenous porphyrin levels (Ramstad et al., 2005), amplifying ROS generation; (ii) increased membrane fluidity at RT renders lipid bilayers more susceptible to peroxidation; and (iii) although enzymatic repair systems (catalase, DNA repair) are also more active at RT, the rate of ROS production exceeds repair capacity, resulting in net greater killing. At 4 °C, reduced metabolism lowers both ROS production and repair, yielding moderate but still significant reductions. These findings are practically relevant because refrigeration is the primary storage condition for many ready-to-eat foods threatened by L. *monocytogenes* breakout; the fact that aBL remains effective at 4 °C (though less so than at RT) supports its use in cold-chain logistics.

A critical aspect of any food decontamination technology is its impact on food quality and consumer safety. We measured key quality parameters (pH, PPO, TBARS, carbonyls, polyphenols) and found no adverse effects; indeed, PPO activity was reduced in orange juice, and textural properties of surimi were improved. However, we acknowledge that other photosensitive nutrients – particularly vitamins A, C, E, and certain carotenoids – were not assessed. Future studies should include targeted analyses of these micronutrients to rule out photo-oxidative degradation. Consumer acceptance is another important consideration; visible light treatment is generally perceived as more “natural” than UV or chemical preservatives and a preliminary consumer test (*n* = 25) showing no significant difference in overall liking between aBL-treated and untreated samples. From a regulatory perspective, aBL is a non-thermal, residue-free physical process, which may facilitate approval under existing frameworks, but specific regional requirements (FDA, EFSA) will require case-by-case validation with standardized protocols.

Compared to established non-thermal technologies, aBL offers several distinct advantages and some limitations. High-pressure processing (HPP) is highly effective but capital-intensive and batch-wise (Moreirinha et al., 2016), whereas aBL can be implemented in continuous flow systems. Pulsed electric fields (PEF) require conductive media and have high energy costs (Pampoukis et al., 2026); aBL works in any transparent medium. Cold plasma generates reactive species that may affect food quality (Naseem et al., 2025), but aBL is milder and more selective. Chemical preservatives (e.g., organic acids, bacteriocins) are cheap but face consumer resistance and residue concerns; aBL is chemical-free. Natural antimicrobial agents, including lysozyme preparations (Naveed et al., 2023; Naveed et al., 2024; Naveed et al., 2025) and biogenic selenium nanoparticles (Si et al., 2024), as well as marine-organism-derived antibacterial and antifungal extracts (Chan, Hussain, Ullah, & Mirani, 2021; Chan, Mirani, Khan, et al., 2021), offer additional biologically based alternatives, though they generally require extraction, formulation, and regulatory approval steps that aBL, as a purely physical process, avoids. The energy consumption of our LED system (20 mW/cm^2^ for 90 min) is moderate, and with advances in LED efficiency, operational costs are decreasing. For scale-up, conveyor-belt systems with high-power LED arrays are commercially available, and our data provide a basis for designing in-package or in-line treatments. However, the need for light penetration limits aBL to transparent foods or surface decontamination, which is a key constraint compared to HPP or PEF that treat bulk volumes. Overall, aBL is best positioned as a complementary hurdle, especially for post-packaging decontamination of transparently packaged products.

Several limitations of this study should be acknowledged. First, it is important to note that all experiments were performed using a single laboratory strain of L. *monocytogenes* BNCC185986. While this strain is a well-characterized reference isolate, it cannot span the genetic and phenotypic diversity among L. *monocytogenes* strains; Second, most aBL treatment assays were conducted with only a single fixed effective dose, without the setup of dose-gradient groups in every test; Third, although transcriptomics reveal the global transcriptional changes upon blue light treatment, functional validations (e.g., gene knockout or complementation) are still needed in future investigations to characterize key stress response pathways; Fourth, the storage stability assessment was limited to a short monitoring period of 48 h, which can then be further prolonged to represent the bona fide storage conditions; Finally, all experimental validations in this study adopted only one type of conventional food packaging material, restricting the generalized application of our research findings in diversified industrial food processing systems.

Based on the findings and limitations of the present study, several future research directions are proposed. First, future studies should evaluate aBL inactivation performance under real-world food contamination scenarios using mixed microbial cultures and naturally contaminated food matrices. Second, extended long-term storage studies are required, as stated in the limitation section. Third, future work should focus on scale-up reactor design and industrial parameter optimization, such as packaging thickness, light transmission rates, and dose attenuation through the film, which should be regarded as a vital downstream procedure to industrial application. Fourth, combining aBL treatment with other mild hurdle technologies is a promising research direction, such as synergistic effects of aBL integrated with mild thermal treatment and natural food antimicrobials, which has been tested in some researches (Manstein Jr. and Silebi 2025).

## 5 Conclusion

In conclusion, this study establishes that 420 nm blue light (aBL) at doses of 108–144 J/cm^2^ achieves 3–4 log reductions in *Listeria monocytogenes* viability across liquid, solid, and packaged food matrices, including under refrigerated conditions, the very environment where this pathogen poses the greatest risk. The bactericidal effect is driven by ROS-mediated oxidative damage, transcriptional disruption of antioxidant and DNA-repair pathways and biofilm disassembly via autolysis-related genes. Importantly, aBL treatment preserved food quality attributes in surimi products, likely due to secondary inhibition of spoilage microflora. These findings collectively validate aBL as a non-thermal, residue-free, and broadly applicable decontamination tool.

For industrial adoption, we recommend 420 nm as the primary wavelength due to its superior bactericidal efficiency over 440 nm. Operationally, aBL should be applied post-packaging to transparent polyethylene films under refrigerated (4 °C) or ambient temperatures, ensuring uniform light distribution. Pre-treatment surface cleaning to remove organic soils is strongly advised, as residual proteins may potentially attenuate light penetration.

Critical knowledge gaps remain and warrant urgent investigation, including systematic testing of packaging materials beyond PE and thickness effects, as well as the impact of light-scattering additives. Long-term storage studies are needed to determine whether aBL-induced sublethal injury leads to resistance formation, and how this affects subsequent refrigeration or shelf-life dynamics. Furthermore, it is important to test validity of aBL against other pathogens and spoilage organisms, particularly involved during surimi storage.

Ultimately, this work moves aBL beyond laboratory proof-of-concept toward a tangible, sustainable solution for food safety. In an era demanding reduced chemical preservatives and lower carbon footprints, blue light offers a physical intervention that aligns with this emerging trend, and its compatibility with existing cold-chain operations and post-packaging treatments provides a distinct advantage over UV-C or thermal pasteurization. It is then recommended to regulators and industry stakeholders to consider aBL as a validated, emerging technology that, with further optimization, could become a standard hurdle in the multi-barrier approach to food preservation.

## CRediT authorship contribution statement

**Biao He:** Writing – original draft, Methodology, Investigation, Data curation. **Yi Xu:** Writing – review & editing, Writing – original draft, Supervision, Methodology, Funding acquisition, Data curation, Conceptualization. **Ruili Yang:** Methodology, Investigation, Conceptualization. **Ying Zhang:** Methodology, Investigation. **Feng Zhu:** Methodology. **Xueqin Cheng:** Investigation. **Jianan Li:** Investigation. **Jun-O Jin:** Writing – review & editing. **Hui-Li Wang:** Writing – review & editing, Funding acquisition, Conceptualization.

## Funding statement

This work was supported by the 10.13039/501100012166National Key Research and Development Program of China (no. 2025YFF1107704), the Open Project of Anhui Key Laboratory of Aerosol Chemistry and Biological Effect (no. M2026308), the Natural Science Foundation of Anhui Province (no. 2508085MC044), the National Natural Science Foundation of China (no. 81673624).

## Declaration of competing interest

The authors declare that they have no known competing financial interests or personal relationships that could have appeared to influence the work reported in this paper.

## Data Availability

Data will be made available on request.
