# Supplementary material for: Antimicrobial blue light inactivates Listeria monocytogenes in diverse food systems with preserved physicochemical and sensory qualities
Source: Food Chem X. 2026 Sep 3;39:104399. doi: 10.1016/j.fochx.2026.104399 (PMC13572314; doi:10.1016/j.fochx.2026.104399)
Supplement: Supplementary file 1 [file mmc1.docx]

**Fig. S1**

**Fig. S1.** Setup used for aBL irradiation. (A) Schematic diagram of the aBL (antimicrobial blue light) irradiation setup, in which the LED source plate was positioned 15 cm above a 6-well plate containing bacterial samples within a closed box. (B) Photograph of the LED source plate emitting blue light.

**Fig. S2**

**Fig. S2.** Representative spot-assay images underlying the wavelength-dependent bactericidal effect of aBL. Representative photographs of spot-assay plates showing colony-forming units of *L. monocytogenes* at serial dilutions (10⁻¹–10⁻⁶) following treatment with aBL at 108 J/cm² across the tested conditions (untreated control and 420, 430, 440, 450, and 460 nm).

**Fig. S3**

**Fig. S3.** aBL induces an antioxidant enzymatic stress response in *Listeria monocytogenes*. (A) Superoxide dismutase (SOD) activity and (B) catalase (CAT) activity in *L. monocytogenes* following 20 min of aBL irradiation at 420 nm or 440 nm (108 J/cm²), measured using commercial activity assay kits (Solarbio Science & Technology, Beijing, China). Data are presented as mean ± SD from eight independent replicates. Statistical significance was determined by one-way ANOVA (*p < 0.05; **p < 0.01; ***p < 0.001).

**Fig. S4**

**Fig. S4.** Original transcriptome-wide gene detection overlap, KEGG enrichment, and porphyrin-pathway gene network for *L. monocytogenes* exposed to aBL, complementing the re-analyzed dataset in Fig. 5. (A) Venn diagram showing the number of genes uniquely detected in the control (Ctrl, 68 genes) and aBL-treated groups (57 genes), and shared between both conditions (2,624 genes) among a total of 2,749 detected genes; this reflects overall transcriptome detection overlap rather than the differentially expressed gene (DEG) set defined in Fig. 5 (|log2FC| > 1, padj < 0.05). (B) Bar chart showing the number of DEGs enriched in the top KEGG pathways. (C) Gene interaction network of porphyrin-metabolism-related genes, with node color representing log2 fold-change values.

**Fig. S5**

**Fig. S5.** Additional physicochemical quality parameters of aBL-treated orange juice, corresponding to Fig. 6. (A) Polyphenol oxidase (PPO) relative activity, (B) soluble solids content (°Brix), and (C) total polyphenol content (mg/mL) of orange juice under the indicated treatment conditions at room temperature (RT, red) and 4°C (green). (D) Consumer overall acceptability scores of orange juice under the indicated treatment conditions at RT and 4°C, assessed by a separate panel of 25 untrained consumers using the same 10-point hedonic scale described in Section 2.12; individual consumer scores are plotted. Data for panels (A–C) are presented as mean ± SEM from three independent experiments. Statistical significance was determined by one-way ANOVA (ns, not significant; *p < 0.05; ***p < 0.001).

**Fig. S6**

**Fig. S6.** Additional textural parameters of aBL-treated sliced surimi, corresponding to Fig. 7. (A) Chewiness, (B) hardness (g), and (C) cohesiveness of sliced surimi under the indicated treatment conditions at RT (red) and 4°C (green). (D) Consumer overall acceptability scores of sliced surimi under the indicated treatment conditions at RT and 4°C, assessed by the separate 25-person consumer panel described in Section 2.15; individual consumer scores are plotted. Data for panels (A–C) are presented as mean ± SEM from three independent experiments. Statistical significance was determined by one-way ANOVA (ns, not significant; *p < 0.05; ***p < 0.001).

**Fig. S7**

**Fig. S7.** Additional textural parameters of aBL-treated packaged surimi, corresponding to Fig. 8. (A) Chewiness, (B) cohesiveness, and (C) hardness (g) of packaged surimi under the indicated treatment conditions at RT (red) and 4°C (green). (D) Consumer overall acceptability scores of packaged surimi under the indicated treatment conditions at RT and 4°C, assessed by the separate 25-person consumer panel described in Section 2.15; individual consumer scores are plotted. Data for panels (A–C) are presented as mean ± SEM from three independent experiments. Statistical significance was determined by one-way ANOVA (ns, not significant; *p < 0.05; **p < 0.01; ***p < 0.001).

| **Wavelength (nm)** | **EC₅₀ (J/cm²)** | **95% CI (J/cm²)** | **EC₉₀ (J/cm²)** | **95% CI (J/cm²)** | **Hill Slope** | **R²** | **Activity Level** |
| --- | --- | --- | --- | --- | --- | --- | --- |
| **420** | **4.2** | 3.5 – 5.1 | **7.5** | 6.3 – 8.9 | −2.3 | 0.995 | Extremely strong |
| **430** | **10.6** | 8.9 – 12.7 | **18.3** | 16.2 – 20.8 | −2.31 | 0.992 | Strong (experimentally fitted) |
| **440** | **52.0** | 45 – 60 | **108.0** | 95 – 122 | −2.3 | 0.988 | Weakest |
| **450** | **28.0** | 24 – 33 | **50.0** | 43 – 58 | −2.3 | 0.990 | Moderate |
| **460** | **22.0** | 19 – 26 | **39.0** | 34 – 45 | −2.3 | 0.991 | Moderately strong |

Table S1 Summary of dose - response fitting parameters for different wavelengths (four-parameter logistic model)

Table S2 Triangle test results of orange juice (n = 16, reference = Ctrl)

| Sample pair | Storage condition | Triangle test correct discriminants | Triangle significant difference (cutoff ≥ 9) | Dominant sensory difference description |
| --- | --- | --- | --- | --- |
| Ctrl vs Mock | RT | 14 | Yes | Severe browning, sour off-flavor, faded citrus aroma, high turbidity |
| Ctrl vs Mock | 4°C | 12 | Yes | Mild browning, slight fermented sourness |
| Ctrl vs aBL (420 nm) | RT | 6 | No | No unified perceivable difference |
| Ctrl vs aBL (420 nm) | 4°C | 5 | No | No unified perceivable difference |

Table S3 Triangle test results of unpackaged surimi (n = 16, reference = Ctrl)

| Sample pair | Storage condition | Triangle test correct discriminants | Triangle significant difference (cutoff ≥ 9) | Dominant sensory difference description |
| --- | --- | --- | --- | --- |
| Ctrl vs Mock | RT | 15 | Yes | Grey surface discoloration, rancid fish off-odor, sticky and soft texture |
| Ctrl vs Mock | 4°C | 13 | Yes | Mild putrid odor, reduced elasticity |
| Ctrl vs aBL (420 nm) | RT | 8 | No | Minor individual feedback of better elasticity, no unified perceivable difference |
| Ctrl vs aBL (420 nm) | 4°C | 7 | No | No unified perceivable difference |

Table S4 Triangle test results of packaged surimi (n = 16, reference = Ctrl)

| Sample pair | Storage condition | Triangle test correct discriminants | Triangle significant difference (cutoff ≥ 9) | Dominant sensory difference description |
| --- | --- | --- | --- | --- |
| Ctrl vs Mock | RT | 14 | Yes | Yellowish surimi, water stain on packaging, obvious spoilage odor |
| Ctrl vs Mock | 4°C | 12 | Yes | Mild abnormal fishy odor, softened texture |
| Ctrl vs aBL (420 nm) | RT | 7 | No | No unified perceivable difference |
| Ctrl vs aBL (420 nm) | 4°C | 5 | No | No unified perceivable difference |
